# The ATPase mechanism of myosin 15, the molecular motor mutated in DFNB3 human deafness

**DOI:** 10.1074/jbc.RA120.014903

**Published:** 2021-01-09

**Authors:** Fangfang Jiang, Yasuharu Takagi, Arik Shams, Sarah M. Heissler, Thomas B. Friedman, James R. Sellers, Jonathan E. Bird

**Affiliations:** 1 Department of Pharmacology and Therapeutics, and the Myology Institute, University of Florida College of Medicine, Gainesville, Florida, USA; 2 Laboratory of Molecular Physiology, Cell and Developmental Biology Center, National Heart, Lung, and Blood Institute, National Institutes of Health, Bethesda, Maryland, USA; 3 Laboratory of Molecular Genetics, National Institute on Deafness and Other Communication Disorders, National Institutes of Health, Bethesda, Maryland, USA; 4 Department of Physiology and Cell Biology, The Ohio State University Wexner Medical Center, Columbus, Ohio, USA

**Keywords:** ATPase, actin, myosin, hearing, hair cell, cytoskeleton, EGFP, enhanced green fluorescent protein, ELC, essential light chain, EPS8, epidermal growth factor receptor kinase substrate 8, LCBD, light-chain binding domain, mantATP, 2′-deoxy-mantATP, MET, mechanoelectric transduction, MOI, multiplicity of infection, MYO15, myosin 15, PEI, polyethylenimine, RLC, regulatory light chain, WHRN, whirlin

## Abstract

Cochlear hair cells each possess an exquisite bundle of actin-based stereocilia that detect sound. Unconventional myosin 15 (MYO15) traffics and delivers critical molecules required for stereocilia development and thus is essential for building the mechanosensory hair bundle. Mutations in the human *MYO15A* gene interfere with stereocilia trafficking and cause hereditary hearing loss, DFNB3, but the impact of these mutations is not known, as MYO15 itself is poorly characterized. To learn more, we performed a kinetic study of the ATPase motor domain to characterize its mechanochemical cycle. Using the baculovirus–*Sf*9 system, we purified a recombinant minimal motor domain (S1) by coexpressing the mouse MYO15 ATPase, essential and regulatory light chains that bind its IQ domains, and UNC45 and HSP90A chaperones required for correct folding of the ATPase. MYO15 purified with either UNC45A or UNC45B coexpression had similar ATPase activities (*k*_cat_ = ∼ 6 s^−1^ at 20 °C). Using stopped-flow and quenched-flow transient kinetic analyses, we measured the major rate constants describing the ATPase cycle, including ATP, ADP, and actin binding; hydrolysis; and phosphate release. Actin-attached ADP release was the slowest measured transition (∼12 s^−1^ at 20 °C), although this did not rate-limit the ATPase cycle. The kinetic analysis shows the MYO15 motor domain has a moderate duty ratio (∼0.5) and weak thermodynamic coupling between ADP and actin binding. These findings are consistent with MYO15 being kinetically adapted for processive motility when oligomerized. Our kinetic characterization enables future studies into how deafness-causing mutations affect MYO15 and disrupt stereocilia trafficking necessary for hearing.

Unconventional myosin 15 (MYO15), encoded by the *MYO15A* gene in humans and *Myo15* in mouse, is a member of the myosin superfamily of P-loop ATPases that generate force on actin filaments (1). MYO15 is expressed by hair cells of the inner ear (2, 3, 4) and is necessary for the structural integrity of actin-based mechanosensory stereocilia that elongate from the surface of hair cells to detect sound and accelerations (4, 5, 6). MYO15 isoforms traffic to the distal tips of stereocilia (4, 7, 8), which is the primary site of actin filament polymerization (9, 10, 11, 12). Mutations in *MYO15A* cause autosomal recessive hearing loss DFNB3 in humans (13, 14, 15), highlighting its critical function in sensory function and the need to understand how this molecular motor operates within stereocilia.

Cochlear hair cells express multiple protein isoforms of MYO15 created through alternative mRNA splicing (2, 4, 8). A short isoform (MYO15-2, also called MYO15-S) contains the core ATPase ‘motor domain’ and three light-chain binding domains (LCBDs) that serve to amplify structural changes within the motor and generate the power stroke (Fig. 1*A*). LCBD sites 1 and 2 are consensus IQ domains that bind to non–muscle regulatory light chain (RLC, MYL6) and essential light chain (ELC, MYL12B), respectively (16). A third LCBD has distant similarity to the IQ consensus sequence; however, no associated light chain has been reported (16). The C-terminal tail of MYO15-2 contains a Src homology 3 domain, and similar to unconventional class VII and X myosins, myosin tail homology 4 and band 4.1, ezrin, radixin, moesin domains, in addition to a PDZ ligand at the C-terminus (2, 17). A larger isoform (MYO15-1, also called MYO15-L) differs from MYO15-2 solely by the addition of a 133-kDa N-terminal domain that is encoded by inclusion of a single exon in the transcript (2) (Fig. 1*A*). No functional domains have been identified within the proline-rich N-terminal domain; however, it is essential for hearing (8).

**Figure 1:**
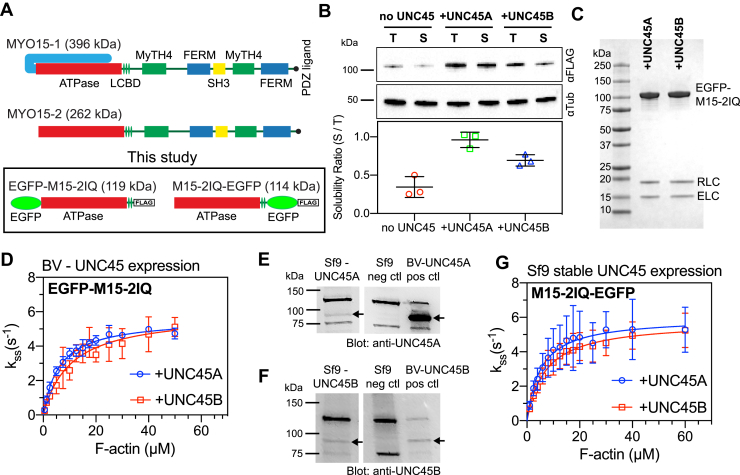
**Purification and steady-state activity of the unconventional myosin 15 (MYO15) motor domain.***A*, drawing of alternative splice isoforms of MYO15 expressed in the inner ear. Each isoform shares a common ATPase domain (*red*) and three light-chain binding domains (LCBDs), in addition to MyTH4 (*green*), SH3 (*yellow*), and FERM (*blue*) domains. Both isoforms are identical, except for the addition of a 133-kDa N-terminal domain in MYO15-1. Baculovirus expression constructs used in this study encode the truncated MYO15 motor domain and two consensus LCBDs fused to enhanced green fluorescent protein (EGFP) and a FLAG tag. *B*, the effect of UNC45 expression upon solubility of the MYO15 motor domain. *Sf*9 cells were infected with baculovirus to express EGFP-M15-2IQ, ELC (MYL6), and RLC (MYL12), plus optionally UNC45A or UNC45B. Representative Western blots (anti-FLAG) of EGFP-M15-2IQ in *Sf*9 cell total lysate (T) *versus* supernatant (S) fractions after sedimentation at 48 kG. Blots were probed for alpha-tubulin as loading controls. A solubility ratio (= S/T) was calculated using densitometry from three independent experimental determinations (data are mean ± SD). Coexpression of either UNC45A (*p* = 0.001) or UNC45B (*p* = 0.017) significantly increased the yield of soluble EGFP-M15-2IQ (one-way ANOVA). *C*, SDS-PAGE analysis of the truncated motor domain (EGFP-M15-2IQ) purified from *Sf*9 cells coexpressing UNC45A or UNC45B. Light chains ELC (MYL6) and RLC (MYL12) are copurified. *D*, steady-state ATPase activation of EGFP-M15-2IQ by actin filaments measured using the NADH assay. Coexpression of EGFP-M15-2IQ with UNC45A (*blue*) yielded *k*_cat_ = 5.6 ± 0.1 s^−1^, *k*_ATPase_ = 6.1 ± 0.5 μM. Coexpression of EGFP-M15-2IQ with UNC45B (*red*) yielded *k*_cat_ = 5.9 ± 0.4 s^−1^, *k*_ATPase_ = 10.4 ± 2.2 μM. Data are from three independent myosin preparations. *E* and *F*, Western blotting of cell lysates from *Sf*9 cells engineered to stably express UNC45A (*E*) or UNC45B (*F*). WT *Sf*9 cell lysates are included as no-UNC45 negative controls, and *Sf*9 cells infected with either UNC45A or UNC45B baculovirus as positive controls. Bands specific for UNC45A + UNC45B are marked with *arrows*. *G*, steady-state ATPase activation of M15-2IQ-EGFP purified from *Sf*9–UNC45A or *Sf*9–UNC45B cell lines, measured using the NADH assay. M15-2IQ-EGFP purified from *Sf*9–UNC45A (*blue*) yielded *k*_cat_ = 6.0 ± 0.5 s^−1^, *k*_ATPase_ = 5.2 ± 1.5 μM. M15-2IQ-EGFP purified from *Sf*9–UNC45B (*red*) yielded *k*_cat_ = 5.8 ± 0.3 s^−1^, *k*_ATPase_ = 7.0 ± 1.3 μM. ATPase data in (*G*) are from three independent determinations, from two independent myosin preparations. All ATPase measurements performed at 20 °C in 10-mM Mops, 10-mM KCl, 5-mM MgCl_2_, 0.1-mM EGTA, 2-mM MgATP, 40 U·mL^−1^ lactate dehydrogenase, 200 U·mL^−1^ pyruvate kinase, 1-mM phosphoenolpyruvate, 200-μM NADH. The basal ATPase activity of myosin was subtracted from each data point. FERM, band 4.1, ezrin, radixin, moesin; MyTH4, myosin tail homology 4; SH3, Src homology 3 domain.

Full-length MYO15-1 and MYO15-2 have independent functions regulating stereocilia architecture in cochlear hair cells. MYO15-2 is sufficient to drive stereocilia elongation during development (8, 17), and in heterologous cell lines, is able to traffic along filopodia similar to class VII and X myosins (4, 17, 18, 19, 20, 21). MYO15-2 complexes with at least four proteins, including whirlin (WHRN), epidermal growth factor receptor kinase substrate 8 (EPS8), guanine nucleotide-binding protein G_i_ subunit alpha, and G-protein signaling modulator 2. MYO15-2 traffics this ‘elongation complex’ along filopodia actin filaments *in vitro*, and *in vivo*, delivers these proteins to the tip compartment of stereocilia, where they are required for polymerization and elongation of the stereocilia actin core (17, 22, 23, 24, 25, 26, 27, 28). The large MYO15-1 isoform is dispensable for stereocilia elongation but is necessary for maintaining the adult length of stereocilia with active mechanoelectric transduction (MET) channels (8). MYO15-1 does not traffic WHRN or EPS8 and its associated cargo proteome is currently unknown (8). The ability of MYO15 isoforms to traffic within stereocilia is thus critical for mechanosensory function, but their mechanisms of motility are poorly understood.

In most members of the myosin superfamily, the motor domain generates force by reversibly binding to actin filaments and undergoing an ATP-dependent mechanochemical cycle. Kinetic tuning of the motor domain significantly diversifies motor behavior on actin filaments and allows myosins to engage in highly specific cellular functions (29, 30, 31, 32). Kinetic tuning of the MYO15 motor domain is currently unknown, yet this information is critical to decipher its function within stereocilia. We previously purified the motor domain of mouse MYO15 and showed it was a fast, high-duty ratio motor that moves toward the barbed end of actin filaments (16). In the present study, we have characterized the motor domain in detail using transient-state kinetic analyses to reveal its key enzymatic adaptations. Our results show that the MYO15 motor domain has kinetic characteristics consistent with sensing strain as a monomer, and that if oligomerized into an ensemble, would be sufficient to enable processive movement along actin filaments.

## Results

### UNC45A and UNC45B each promote folding of the MYO15 motor domain

UNC45 is required for the correct folding and assembly of muscle thick filaments (33) and acts as a HSP90-dependent co-chaperone that folds the muscle myosin motor domain (34, 35). We and others have reported that UNC45 also catalyzes the folding of some non–muscle myosin motor domains when expressed in the *Sf*9 system (16, 36), indicating that UNC45 acts more broadly to fold myosin motors. Mammals express two UNC45 paralogs, with 57% amino acid identity in humans. UNC45A is expressed ubiquitously throughout the body, while UNC45B is more restricted to striated muscle tissues although not solely expressed there (37). Interestingly, cochlear hair cells express both UNC45A and UNC45B (https://umgear.org), raising the possibility that both co-chaperones might be used to fold MYO15 *in vivo*.

Our previous work demonstrated that coexpression of UNC45B significantly increased the yield of purified, enzymatically active MYO15 motor domain when produced in *Sf*9 insect cells (16). We tested whether UNC45A could catalyze folding of the MYO15 motor domain similar to the action of UNC45B. To do this, a minimal motor domain construct (S1-like) of mouse MYO15 (abbreviated EGFP-M15-2IQ) that contained the ATPase plus two LCBDs was expressed in *Sf*9 insect cells using the baculovirus expression system. An enhanced green fluorescent protein (EGFP) moiety and FLAG epitope were fused to the N-terminus and C-terminus of the motor domain, respectively (Fig. 1*A*). RLC and ELC were coexpressed to bind the LCBD (16). In addition to MYO15 and light chains, *Sf*9 cells were also infected with a dual-promoter baculovirus expressing HSP90AA1 and either UNC45A or UNC45B (see Experimental procedures).

To test the relative activities of UNC45 isoforms, small-scale *Sf*9 cell cultures (200 ml) were infected with EGFP-M15-2IQ plus light chains, and optionally with (1) HSP90AA1/UNC45A or (2) HSP90AA1/UNC45B. A ‘no UNC45’ control experiment was run in parallel where no UNC45 isoforms were expressed. After 48 h, whole cell lysates from infected *Sf*9 cells were sedimented at 48-kG (see Experimental procedures) and the total (T) and supernatant (S) fractions analyzed by Western blotting (Fig. 1*B*). We calculated a solubility ratio, defined as the amount of EGFP-M15-2IQ in the supernatant fraction, relative to the total cell lysate (solubility = S/T). Solubility ratios for EGFP-M15-2IQ with no UNC45, or coexpressed with UNC45A, or UNC45B were 0.35 ± 0.14, 0.96 ± 0.10, and 0.69 ± 0.07, respectively (data are mean ± SD from three independent determinations) (Fig. 1*B*). Coexpression with UNC45A (*p* = 0.001), and separately UNC45B (*p* = 0.017), significantly increased the solubility of EGFP-M15-2IQ relative to the no UNC45 control (one-way ANOVA). These results agree with our previous study that coexpression with UNC45B can improve solubility of the MYO15 motor domain (16) and extend them to show that UNC45A has a similar activity.

Supernatants from these expressions were further purified by FLAG affinity chromatography, before eluted EGFP-M15-2IQ was bound to an anion exchanger (Mono Q). We quantitatively eluted EGFP-M15-2IQ with a NaCl gradient (see Experimental procedures) and measured the area under the EGFP-M15-2IQ chromatogram peak to give total yields of 75.8 ± 13.8 mAU (for UNC45A) *versus* 15.1 ± 7.1 mAU (for UNC45B). This demonstrated a statistically significant (*p* = 0.007, two-tailed *t*-test, three independent determinations) increase in myosin yield using UNC45A *versus* UNC45B. Only trace quantities of EGFP-M15-2IQ were eluted in the no-UNC45 sample, consistent with our previous findings (16). EGFP-M15-2IQ purified with either UNC45A or UNC45B was indistinguishable by SDS-PAGE (Fig. 1*C*). We conclude that both UNC45A and UNC45B catalyze folding of the MYO15 motor domain in *Sf*9 cells but that UNC45A does so with a higher efficiency.

We next hypothesized that the activity of the motor domain might be influenced by the specific UNC45 chaperone recruited to fold MYO15. To test this, the steady-state ATPase activity of EGFP-M15-2IQ, purified from *Sf*9 cells coexpressing either UNC45A or UNC45B, was measured using an enzyme-linked NADH assay (Fig. 1*D*). The apparent affinity of EGFP-M15-2IQ for actin in the presence of ATP is strongly dependent upon the salt concentration (16), and our assays were performed with 10-mM KCl to increase the affinity of EGFP-M15-2IQ for actin. The basal ATPase rates measured in the absence of actin were 0.05 ± 0.01 s^−1^ (UNC45A) and 0.06 ± 0.01 s^−1^ (UNC45B) and were not significantly different (*p* = 0.17, two-tailed *t*-test). Actin was next titrated into the reaction to measure actin-activated ATPase activity. ATPase rates were fit to a hyperbola to estimate *k*_cat_ = 5.6 ± 0.1 s^−1^ and *K*_ATPase_ = 6.1 ± 0.5 μM for EGFP-M15-2IQ purified from *Sf*9 cells coexpressing UNC45A. The *k*_cat_ reflects the maximum catalytic activity, while *K*_ATPase_ is the concentration of actin required for half-maximal activation of ATPase activity. Identical measurements were performed for EGFP-M15-2IQ purified from *Sf*9 cells expressing UNC45B, yielding *k*_cat_ = 5.9 ± 0.4 s^−1^ and *K*_ATPase_ = 10.4 ± 2.2 μM. Comparison between UNC45A and UNC45B revealed no statistically significant change to either *k*_cat_ (*p* = 0.62) or *K*_ATPase_ (*p* = 0.10, two-tailed *t*-test). Key ATPase parameters are summarized in Table 1. We conclude that coexpression of either UNC45 paralog is sufficient to produce enzymatically active EGFP-M15-2IQ and that the use of UNC45A *versus* UNC45B did not affect the overall ATPase activity. To be consistent with our previous work (16), UNC45B coexpression has been used for all EGFP-M15-2IQ transient kinetics experiments described in this study.

**Table 1:** Summary of steady-state ATPase measurements

Protein	*k*_cat_ (s^−1^)	*k*_ATPase_ (μM)	Basal ATPase (s^−1^)
EGFP-M15-2IQ (baculovirus UNC45A)	5.6 ± 0.1	6.1 ± 0.5	0.05 ± 0.01
EGFP-M15-2IQ (baculovirus UNC45B)	5.9 ± 0.4	10.4 ± 2.2	0.06 ± 0.01
M15-2IQ-EGFP (*Sf*9 - UNC45A)	6.0 ± 0.5	5.2 ± 1.5	0.1 ± 0.01
M15-2IQ-EGFP (*Sf*9 - UNC45B)	5.8 ± 0.3	7.0 ± 1.3	0.09 ± 0.01

Experimental conditions: 10-mM Mops, pH 7.0, 10-mM KCl, 5-mM MgCl_2_, 0.1-mM EGTA, 2-mM MgATP at 20 ± 0.1 °C. Data are the mean ± SEM (for *k*_cat_ and *k*_ATPase_) and mean ± SD (for basal ATPase).

### Development of Sf9–UNC45A and Sf9–UNC45B cell lines to streamline MYO15 motor domain purification

To simplify the expression of the MYO15 motor domain, and potentially other myosin motor domains that require UNC45 to fold, we developed clonal *Sf*9 cell lines that constitutively express UNC45A or UNC45B (see Experimental procedures). WT *Sf*9 cells do not natively express either of these chaperones but do express HSP90 orthologs (16, 38). Total protein extracts from clonal *Sf*9-UNC45A and *Sf*9-UNC45B cell cultures were screened by SDS-PAGE and Western blotting to confirm the expression of UNC45A or UNC45B, respectively (arrows in left lanes, Fig. 1, *E* and *F*). WT *Sf*9 cell lysates were used as negative controls. *Sf*9 cells infected with baculovirus to overexpress UNC45A/HSP90AA1 or UNC45B/HSP90AA1 served as positive controls (arrows in right lane, Fig. 1, *E* and *F*). To test if these cell lines were functional for expressing MYO15, *Sf*9–UNC45A and *Sf*9–UNC45B cells were infected with baculovirus encoding the motor domain with a C-terminal EGFP and FLAG moiety (Fig. 1*A*, M15-2IQ-EGFP). *Sf*9 cells were additionally coinfected with baculovirus encoding the ELC and RLC. Purified M15-2IQ-EGFP from either cell line had similar actin-activated ATPase activities. Basal ATPase rates measured in the absence of actin were 0.1 ± 0.01 s^−1^ (*Sf*9–UNC45A) and 0.09 ± 0.01 s^−1^ (*Sf*9–UNC45B) and were not significantly different (*p* = 0.72, two-tailed *t*-test). M15-2IQ-EGFP purified from *Sf*9–UNC45A cells exhibited *k*_cat_ = 6.0 ± 0.5 s^−1^ and *K*_ATPase_ = 5.2 ± 1.5 μM, while M15-2IQ-EGFP purified from *Sf*9–UNC45B had *k*_cat_ = 5.8 ± 0.3 s^−1^ and *K*_ATPase_ = 7.0 ± 1.3 μM (see Fig. 1*G*, Table 1). Comparison between M15-2IQ-EGFP activity purified from *Sf*9–UNC45A or *Sf*9–UNC45B revealed no statistically significant difference in *k*_cat_ (*p* = 0.86) or *K*_ATPase_ (*p* = 0.13, two-tailed *t*-test). These results corroborate our findings that the ATPase activity of MYO15 does not differ between the usage of UNC45A *versus* UNC45B and further shows that the *Sf*9–UNC45A and *Sf*9–UNC45B cells lines are effective at expressing enzymatically active myosin motor domains.

### Interaction of MYO15 with ATP

**Figure 2:**
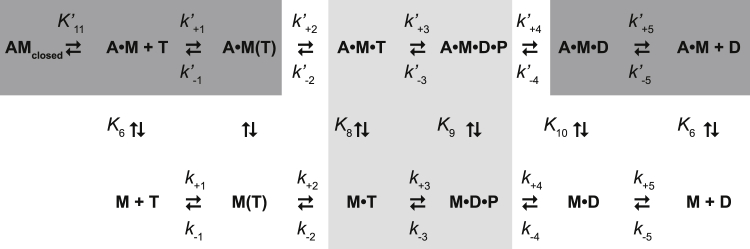
**ATPase mechanism of the MYO15 motor domain.** Species are abbreviated as follows: actin (A), myosin (M), ATP (T), ADP (D) and inorganic phosphate (P). Strongly actin-bound species (*dark gray*) and weakly actin-bound species (*light gray*). Equilibrium constants are defined as *K*_i_ = *k*_+i_/*k*_−i_, where the *k*_+i_ reaction proceeds up or to the right. Actin-bound state transitions are denoted with an apostrophe (*e.g.*, *k*′_+2_).

**Figure 3:**
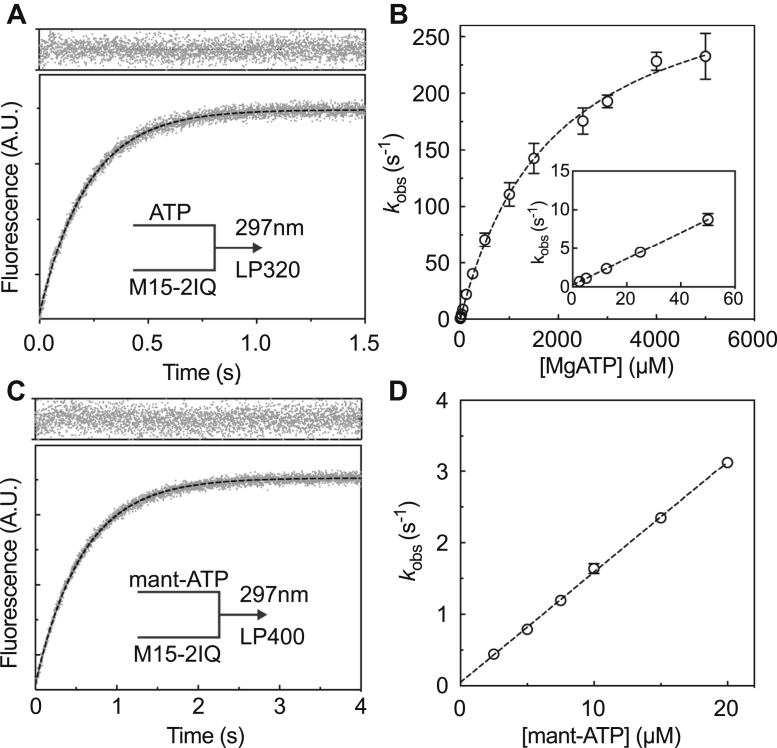
**Transient kinetic analysis of ATP binding to EGFP-M15-2IQ using stopped-flow fluorescence spectroscopy.***A*, intrinsic fluorescence enhancement as 0.25-μM EGFP-M15-2IQ was rapidly mixed with 25-μM ATP. The transient was fit to a single exponential equation, *I*(*t*) = −9.2·*e*^−4.52*t*^ + *C* (*dotted line*, residuals above). *B*, dependence of intrinsic fluorescence observed rate constants (*k*_obs_) upon ATP concentration. The hyperbola *k*_obs_ = (*K*_1_·*k*_+2_·[ATP])/(1 + *K*_1_·[ATP]) is shown (*dotted line*), where 1/*K*_1_ = 1898 ± 145 μM, *k*_+2_ = 322 ± 10 s^−1^. Inset: *k*_obs_ is shown at lower [ATP]. ATP binding was irreversible. *C*, fluorescence enhancement of 10-μM mant-ATP after rapid mixing with 0.25-μM EGFP-M15-2IQ. The mant fluorophore was excited by FRET from vicinal tryptophan residues. The transient was fit to a single exponential equation *I*(*t*) = −5.9 *e*^−1.71*t*^ + *C* (*dotted line*, residuals above). *D*, dependence of *k*_obs_ upon mant-ATP concentration reveals the apparent association rate constant *K*_1_·*k*_+2_ = 0.15 ± 0.002 μM^−1^ s^−1^ with an intercept of ∼0, showing irreversible binding of mant-ATP to EGFP-M15-2IQ. For all experiments, conditions in the observation cell were 20-mM Mops (pH 7.0), 100-mM KCl, 5-mM MgCl_2_, and 0.1-mM EGTA at 20 °C. Concentrations are postmixing in the observation cell. Experimental data were measured from three independent myosin preparations. mantATP, 2′-deoxy-mantATP.

To measure the kinetic and equilibrium constants describing the ATPase cycle of EGFP-M15-2IQ (see Fig. 2), we performed a series of pre–steady-state experiments using stopped-flow and quenched-flow techniques. ATP generated a robust increase in intrinsic EGFP-M15-2IQ protein fluorescence excited at 297 nm in a stopped-flow spectrophotometer, and we used this property to measure the ATP binding mechanism. EGFP-M15-2IQ (0.25 μM) was rapidly mixed under pseudo–first-order conditions with increasing concentrations of [ATP] ranging up to 5 mM. Experimentally recorded fluorescence transients were fit to a monophasic exponential increase (Fig. 3*A*) and observed rate constants (*k*_obs_) varied hyperbolically with respect to [ATP] (Fig. 3*B*). This reaction was modeled using a two-step binding mechanism (Equation 1), where ATP and myosin form a collision complex in rapid equilibrium (*K*_1_), followed by an isomerization (*k*_+2_) to an enhanced fluorescence state (M∗⋅ATP).

(1) $$\text{M}+\text{ATP}\overset{K_{1}}{⇌}\text{M}\left(\text{ATP}\right)\overset{k_{+2}}{\to }{\text{M}}^{\text{∗}}\cdot \text{ATP}$$

The apparent association rate constant (*K*_1_⋅*k*_+2_) for ATP binding was 0.17 ± 0.005 μM^−^^1^s^−1^, measured from the initial gradient at low [ATP] (Fig. 3*B*, inset). A hyperbolic fit to the intrinsic fluorescence data yielded estimates for the ATP dissociation constant 1/*K*_1_ = 1898 ± 145 μM and isomerization rate constant *k*_+2_ = 322 ± 10 s^−1^ (Fig. 3*B*). Our data indicated that ATP binding was effectively irreversible within the range of measurement uncertainty (*e.g.*, *k*_−2_ ∼ 0). Mouse MYO15 has a conserved tryptophan (W432) in the relay loop, homologous to W501 in conventional myosin (MhcA, *Dictyostelium discoideum*) and W512 in smooth muscle myosin (*Gallus gallus*). In conventional myosin, this residue is proposed to sense conformational changes occurring with ATP hydrolysis (39, 40). Because the exact contribution of W432 to the intrinsic fluorescence signal of MYO15 is unclear, we attribute the maximum observed rate to represent nucleotide binding (*k*_+2_).

ATP binding was independently assessed using the fluorescent nucleotide analog, 2′-deoxy-mantATP (mantATP). FRET from vicinal tryptophan residues was used to excite mantATP as it bound to EGFP-M15-2IQ. EGFP-M15-2IQ (0.25 μM) was rapidly mixed under pseudo–first-order conditions with mantATP titrated from 2.5 μM to 20 μM in the stopped flow. Fluorescence transients were well fit by a monophasic exponential increase (Fig. 3*C*), with observed rate constants (*k*_obs_) varying linearly with [ATP] (Fig. 3*D*). Linear regression to the observed rate constants yielded the association rate constant, *K*_1_⋅*k*_+2_ = 0.15 ± 0.002 μM^−^^1^s^−1^, similar to that measured using intrinsic fluorescence.

**Figure 4:**
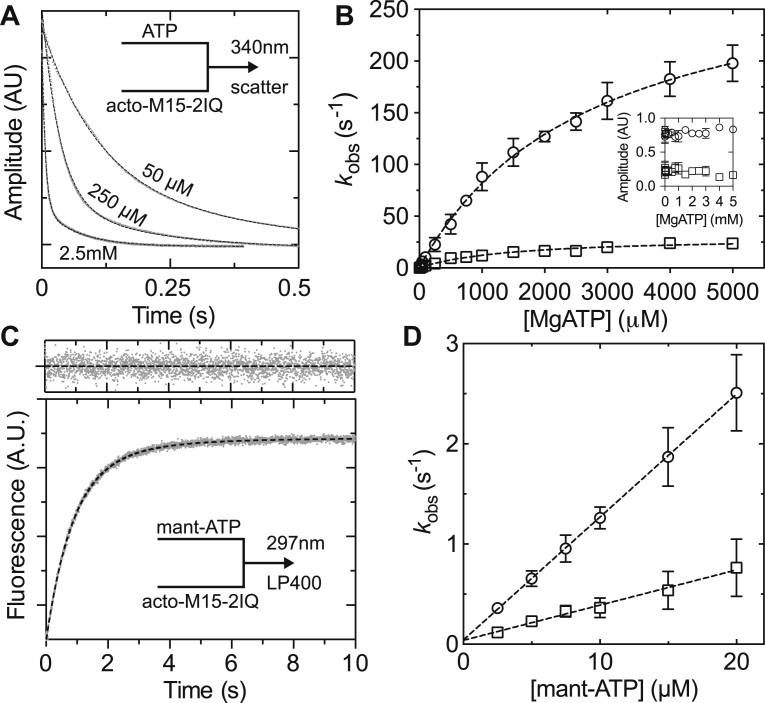
**Transient kinetic analysis of ATP binding to actomyosin using stopped-flow spectroscopy.***A*, reduction in orthogonal light scattering (measured at 340 nm) as 50-, 250-, or 2500-μM MgATP bound to a preincubated mix of 0.25-μM EGFP-M15-2IQ plus 0.5-μM actin in the stopped flow. Transients were fit to a biphasic exponential (*dotted lines*) with *I*(*t*) = 36.2·*e*^−8.7*t*^ + 13.3·*e*^−1.7*t*^ + *C*, *I*(*t*) = 42.2·*e*^−28.7*t*^ + 10.1·*e*^−5.3*t*^ + *C*, *I*(*t*) = 37.5·*e*^−150.6*t*^ + 8.4·*e*^−14.7*t*^ + *C*, for 50-, 250-, and 2500-μM MgATP, respectively. *B*, dependence of fast and slow observed rate constants upon [MgATP]. Hyperbolae were fit (*dotted lines*) to both fast (*circle*) and slow (*square*) phases to yield maximum values of 307 ± 15 s^−1^ and 30.9 ± 1.9 s^−1^, with half maximal activation at 2749 ± 268 μM and 1428 ± 225 μM, respectively. Inset: normalized amplitudes of fast (*circle*) and slow (*square*) phases at different [ATP]. *C*, fluorescence enhancement of 10-μM mant-ATP after rapid mixing with 0.25-μM EGFP-M15-2IQ + 0.5 μM actin. The mant fluorophore was excited by FRET from vicinal tryptophan residues in EGFP-M15-2IQ. The transient was fit to a biphasic exponential *I*(*t*) = −5.0·*e*^−1.2*t*^ – 0.9·*e*^−0.4*t*^ + *C* (*dotted line*, fit residuals above). *D*, dependence of fast and slow observed rate constants upon [mant-ATP]. A linear fit to the fast phase (*circles*) yields the apparent association rate constant *K*′_1_·*k*′_+2_ = 0.12 ± 0.01 μM^−1^ s^−1^ with an intercept of ∼0, showing irreversible binding of mant-ATP to actomyosin. Conditions in the observation cell were 20-mM Mops (pH 7.0), 100-mM KCl, 5-mM MgCl_2_, and 0.1-mM EGTA at 20 °C. All concentrations are postmixing in the observation cell. Experimental data were measured from three independent myosin preparations. mantATP, 2′-deoxy-mantATP.

We next measured ATP-induced dissociation of the actomyosin complex using the decrease of orthogonally scattered light in a stopped-flow experiment. Nucleotide-free EGFP-M15-2IQ (0.125 μM) pre-equilibrated with actin filaments (0.25 μM) was rapidly mixed with [ATP] under pseudo–first-order conditions. Light scattering was followed at 340 nm to monitor dissolution of the actomyosin complex. At [ATP] < 5 μM, observed transients followed a monophasic exponential time course; however, at [ATP] > 12.5 μM, transients were fit to a biphasic exponential decay with clearly defined fast and slow rates (Fig. 4*A*). A two-step serial binding mechanism cannot be used to model this response because both fast and slow observed rate constant phases saturated at higher [ATP] (Fig. 4*B*). Similar kinetics of ATP binding to actomyosin have been reported for MYO1B and MYO19 (41, 42, 43). Following those studies, we interpret these data according to Equation 2, where nucleotide-free actomyosin is in equilibrium (*K*_α_) between a nucleotide-insensitive (AM′) and nucleotide-sensitive (AM) state competent to bind ATP (41). In the nucleotide-sensitive state, ATP and actomyosin form a collision complex in rapid equilibrium (*K*_1_′) that isomerizes (*k*′_+2_ + *k*′_−2_) to an AM⋅ATP state that rapidly dissociates (*K*_8_) from actin. Population of the A + M⋅ATP state results in a decrease in light scattering.

(2) $${\text{AM}}^{\prime }\overset{k_{\alpha }^{\prime }}{\underset{k_{-\alpha }^{\prime }}{⇌}}\text{AM}\;+\;\text{ATP}\overset{K_{1}^{\prime }}{⇌}\text{AM}\cdot \left(\text{ATP}\right)\overset{k_{+2}^{\prime }}{\underset{k_{-2}^{\prime }}{\to }}\text{AM}\cdot \text{ATP}\overset{K_{8}}{⇌}\text{A}\;+\;\text{M}\cdot \text{ATP}$$

The fast phase (*k*_fast_) was interpreted as direct binding of ATP to the nucleotide-sensitive (AM) state (Fig. 4*B*), and the apparent association rate constant was estimated from the initial gradient of this response at low [ATP], *K*′_1_⋅*k*′_+2_ = 0.12 ± 0.02 μM^−1^s^−1^. A hyperbolic fit to *k*_fast_ yielded the maximum isomerization rate *k*′_+2_ = 306.8 ± 15.1 s^−1^ (*k*′_−2_ was ∼0 within the measurement uncertainty) and 1/*K*′_1_ = 2749 ± 268 μM (Fig. 4*B*). The slow observed phase represented *k*′_α_, the transition rate from the nucleotide-insensitive (AM′) to sensitive binding state (AM). This interpretation is valid at high [ATP], where the condition *K*′_1_⋅*k*′_+2_[ATP] >> *k*′_−α_ is satisfied. We estimated the maximum rate *k*′_α_ = 30.9 ± 1.9 s^−1^ using a hyperbolic fit to the *k*_slow_ second-order response (Fig. 4*B*). The equilibrium constant (*K*′_α_) was estimated from the relative amplitudes of the fast and slow phases, which represent the respective fraction of actomyosin in nucleotide-sensitive/nucleotide-insensitive states, *K*′_α_ = A_fast_/A_slow_ = 3.8 ± 1.0 (Fig. 4*B*, inset).

ATP binding to actomyosin was independently probed using mant-ATP fluorescence excited by FRET from vicinal tryptophan residues in EGFP-M15-2IQ. Nucleotide-free EGFP-M15-2IQ (0.25 μM) was preincubated with actin filaments (0.5 μM) and rapidly mixed with mantATP under pseudo–first-order conditions in the stopped flow. Similar to our light-scattering measurements, fluorescence transients were well fit to a biphasic exponential increase (Fig. 4*C*) up to the maximum mant-ATP concentration probed (20 μM). Both observed rate constants varied linearly with [ATP], and a linear regression fit to the fast phase was interpreted as the apparent association rate constant of binding to the nucleotide-sensitive fraction, *K*′_1_⋅*k*′_+2_ = 0.12 ± 0.01 μM^−1^ s^−1^, in exact agreement with our estimate using light scattering (Fig. 4*D*).

### ATP hydrolysis and actin-activated phosphate release from MYO15

**Figure 5:**
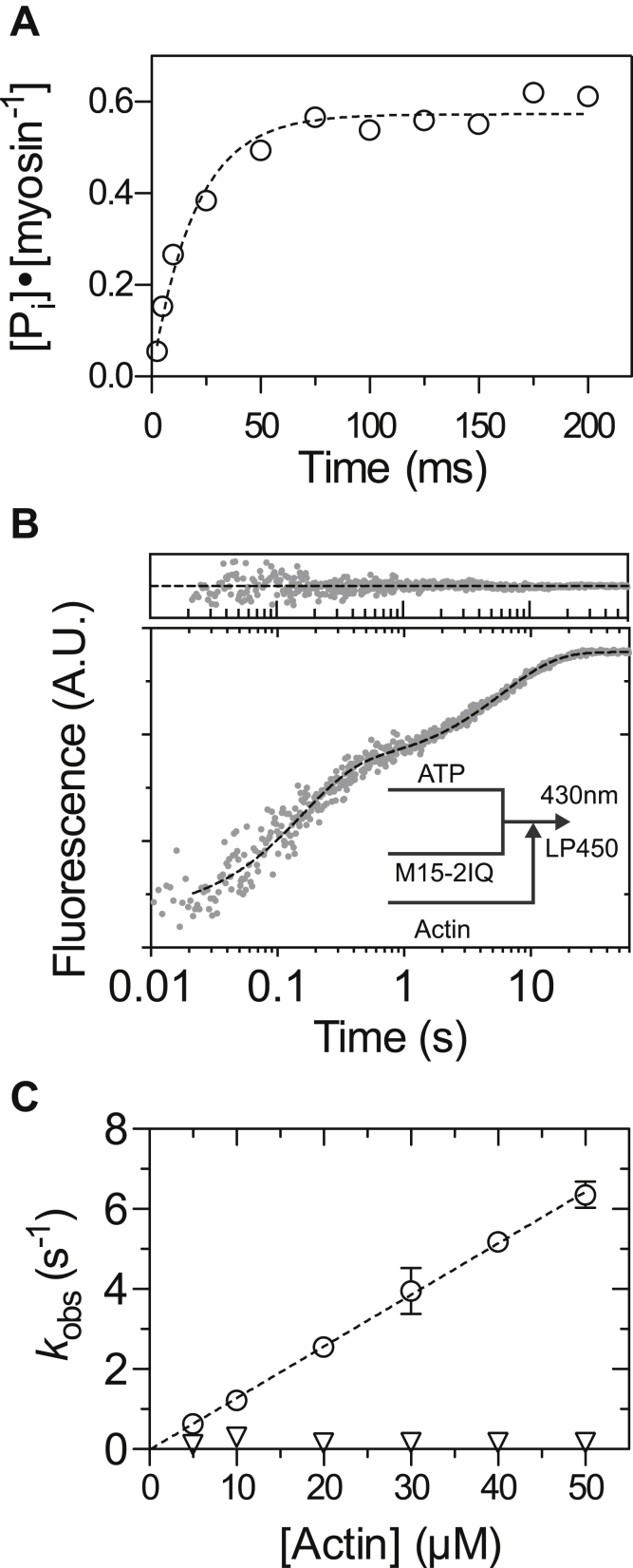
**Hydrolysis and actin-activated phosphate release.***A*, ATP hydrolysis measured by quenched-flow. EGFP-M15-2IQ (3 μM) was reacted under multiple turnover conditions with 0.5-mM [γ-^32^P]ATP and aged for varying intervals before acid quench. The observed phosphate burst was fit to a single exponential, *I*(*t*) = (*K*_*3*_*/1 + K*_*3*_)·(1 − *e*^−*kobs*·*t*^) to yield *k*_obs_ = 50.2 ± 5.7 s^−1^ and *K*_3_ = 1.34 ± 0.07. Data are from one myosin preparation. *B*, double-mixing stopped flow experiment to measure actin-activated phosphate release during a single turnover. EGFP-M15-2IQ (2 μM) was 1:1 mixed with 1-μM ATP and aged for 5 s (postmix concentrations), followed by a subsequent 1:1 mix with actin (final condition in reaction cell, 1-μM myosin, 0.5-μM ATP, 50-μM actin). MDCC-PbP was included in all solutions at 5 μM. Transient is shown overlaid with *I*(*t*) = −1.39*e*^−6.59*t*^ – 1.06*e*^−0.17*t*^ + *C* (*dotted line*, fit residuals above). *C*, dependence of observed rate constants upon [actin]. A linear fit to the fast phase (*circles*) yields *K*_9_·*k*′_+4_ = 0.13 ± 0.004 μM^−1^ s^−1^. The slow observed phase (*triangles*) did not vary systematically with [actin]. Data are from two independent determinations, from one myosin preparation. Conditions in the reaction loop/observation cell were 20-mM Mops (pH 7.0), 100-mM KCl, 5-mM MgCl_2_, 0.1-mM EGTA at 20 °C.

The rate of ATP hydrolysis by EGFP-M15-2IQ in the absence of actin filaments was measured directly in a multiple-turnover quenched-flow experiment. EGFP-M15-2IQ (3 μM) was rapidly mixed under pseudo–first-order conditions with 0.5-mM [gamma-^32^P]-ATP and aged for increasing periods of time, before acid quench and determination of hydrolyzed P_i_ liberated during the reaction. The measured P_i_-burst was well fit by a single exponential with *k*_obs_ = 50.2 ± 5.7 s^−1^ (Fig. 5*A*), representing the net flux of actin-detached forward and reverse hydrolysis (*k*_obs_ = *k*_+3_ + *k*_−3_). The amplitude of the P_i_-burst was 0.57 mol P_i_: mol myosin (Fig. 5*A*), and the equilibrium constant (*K*_3_) was calculated using the relation 0.57 = *K*_3_/(1 + *K*_3_), *K*_3_ = 1.34 ± 0.07 (44). Our data indicate that EGFP-M15-2IQ undergoes significant reverse hydrolysis and that the M⋅ATP and M⋅ADP⋅P_i_ states are both significantly populated at steady state. The observed rate of hydrolysis in this multiple-turnover experiment was comparable with the rate of ATP binding measured using intrinsic fluorescence at an equivalent [ATP] (Fig. 3, *A* and *B*), demonstrating that hydrolysis was likely rate limited by ATP binding in this experiment. Although we likely did not probe the maximum rate of hydrolysis, our data show that this transition does not rate-limit the catalytic cycle of EGFP-M15-2IQ.

Actin-activated P_i_ release from EGFP-M15-2IQ was examined using a single-turnover, dual-mixing stopped-flow experiment. EGFP-M15-2IQ (2 μM) was rapidly mixed with a substoichiometric quantity of ATP (1 μM) and aged for 5 s to allow for hydrolysis. The aged reaction was then rapidly mixed with actin filaments to accelerate P_i_ release from the AM⋅ADP⋅P_i_ quaternary complex. A fluorescent, coumarin labeled phosphate-binding protein (MDCC-PbP) was included in all solutions to monitor P_i_ release in real-time (45). In the absence of actin filaments in the second mix, P_i_ release followed a single exponential time course with *k*_obs_ = 0.08 ± 0.001 s^−1^. This rate is comparable to the steady-state ATPase activity of EGFP-M15-2IQ measured in the absence of actin (see Table 1) and confirms that P_i_ release rate-limits the basal ATPase cycle in the absence of actin filaments. When actin filaments were introduced into the second mix to accelerate P_i_ release, we observed a biphasic exponential increase in the MDCC-PbP signal (Fig. 5*B*). The fast-observed rate (*k*_fast_) varied linearly with [actin], while the slow-observed rate (*k*_slow_) was insensitive to [actin] and remained constant at ∼ 0.16 s^−1^ (Fig. 5*C*). The fast phase was interpreted as P_i_ release upon direct binding of M⋅ADP⋅P_i_ to actin, while the slower phase may originate from the equilibrium of prehydrolysis M⋅ATP species that undergo actin-attached hydrolysis (M⋅ATP => AM⋅ATP => AM⋅ADP⋅P_i_) that subsequently rate-limits P_i_ release (46, 47, 48). A linear regression fit to the *k*_fast_ second-order response yielded an apparent association rate constant, *K*_9_⋅*k*′_+4_ = 0.13 ± 0.004 μM^−1^s^−1^, indicating weak affinity of the M⋅ADP⋅P_i_ species for actin. There was no evidence of saturation in the fast-observed phase that would allow the maximum rate of P_i_ release (*k*′_+4_) to be determined. Given the fastest P_i_ release measured here (∼6 s^−1^ at 50-μM actin, 100-mM KCl) was already four times faster than the equivalent steady-state activity previously reported (∼1.5 s^−1^) under identical buffer conditions (50-μM actin, 100-mM KCl) (16), we conclude that P_i_ release does not rate-limit the catalytic cycle of EGFP-M15-2IQ in the presence of actin.

### The interaction of MYO15 with actin filaments

**Figure 6:**
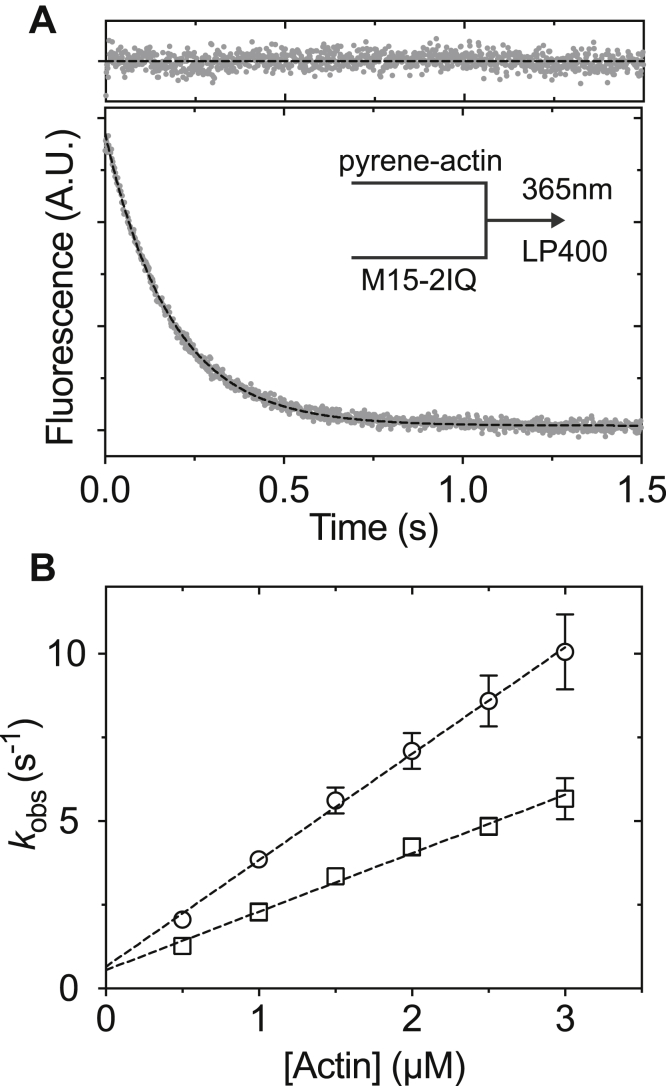
**The interaction of EGFP-M15-2IQ with actin filaments.***A*, reduction in fluorescence measured as 0.15-μM EGFP-M15-2IQ binds and quenches 1.5-μM pyrene-labeled actin in a stopped-flow spectrophotometer in the absence of nucleotide (apyrase-treated). The transient was fit to a single exponential function *I*(*t*) = 5.6*e*^−5.4*t*^ + *C* (*dotted line*, fit residuals above). Similar experiments were repeated in the presence of 1-mM ADP (not shown). *B*, dependence of *k*_obs_ upon [actin] is shown for nucleotide-free conditions (*circles*) and with 1-mM ADP present (squares). Linear regression to *k*_obs_ = *k*_+A_[actin] + *k*_−A_ yields the association rate constant for actin binding *k*_+6_ = 3.18 ± 0.15 μM^−1^s^−1^ and off-rate *k*_−6_ = 0.65 ± 0.3 s^−1^. In the presence of ADP, *k*_+10_ = 1.75 ± 0.09 μM^−1^s^−1^ and *k*_−10_ = 0.55 ± 0.17 s^−1^. Experimental conditions in observation cell: 0.05- to 0.3-μM EGFP-M15-2IQ, 0.5- to 3-μM pyrene-actin, 1-mM ADP (optional), 20-mM Mops (pH 7.0), 100-mM KCl, 5-mM MgCl_2_, 0.1-mM EGTA at 20 °C. Experimental data were measured from three independent myosin preparations.

A stopped-flow fluorescence assay was used to measure binding of EGFP-M15-2IQ to actin in the absence of ATP. We used the quenching of pyrene-iodoacetamide coupled to Cys374 of actin as a probe for the formation of the strongly bound actomyosin state (49). Nucleotide-free conditions were ensured by the pretreatment of actin and myosin solutions with apyrase. EGFP-M15-2IQ (ranging from 0.05 to 0.3 μM) was rapidly mixed with an excess of pyrene actin (ranging from 0.5 to 3 μM) under pseudo–first-order conditions in the stopped-flow spectrophotometer. We held the myosin-to-actin ratio constant throughout the titration to ensure sufficient signal-to-noise at higher actin concentrations. The time course of pyrene fluorescence quenching was fit to a monophasic exponential decay (Fig. 6*A*) with observed rate constants (*k*_obs_) that varied linearly with respect to [actin] (Fig. 6*B*). These data were interpreted using a one-step binding mechanism, and linear regression fit to *k*_obs_ = *k*_+6_[actin] + *k*_−6_ yielded the apparent association rate constant *k*_+6_ = 3.18 ± 0.15 μM^−1^ s^−1^. The apparent dissociation rate *k*_−6_ = 0.65 ± 0.3 s^−1^ was measured from the *y*-axis intercept (Fig. 6*B*). These experiments were repeated in the presence of 0.5-mM ADP to saturate the EGFP-M15-2IQ nucleotide binding site (apyrase was omitted in these experiments). Fluorescence transients in the presence of ADP were well modeled by a monophasic exponential decay, and the observed rate constant again varied linearly with [actin] (Fig. 6*B*). Linear regression to this response yielded the apparent association rate constant *k*_+10_ = 1.75 ± 0.09 μM^−1^ s^−1^ and apparent dissociation rate *k*_−10_ = 0.55 ± 0.17 s^−1^. Dissociation equilibrium constants for EGFP-M15-2IQ binding to actin were calculated using the relation at equilibrium 1/*K*_6_ = *k*_−6_/*k*_+6_, yielding *K*_A_ = 1/*K*_6_ = 200 ± 100 nM under nucleotide-free conditions and *K*_DA_ = 1/*K*_10_ = 310 ± 97 nM in the presence of saturating ADP. We conclude that EGFP-M15-2IQ has a moderate affinity for actin in the absence of ATP. The ratio of actin affinity in the presence and absence of ADP, *K*_DA_/*K*_A_ = 1.6, indicating weak thermodynamic coupling between actin and ADP binding to EGFP-M15-2IQ.

### ADP release from acto-MYO15 is the slowest step in the ATPase cycle

**Figure 7:**
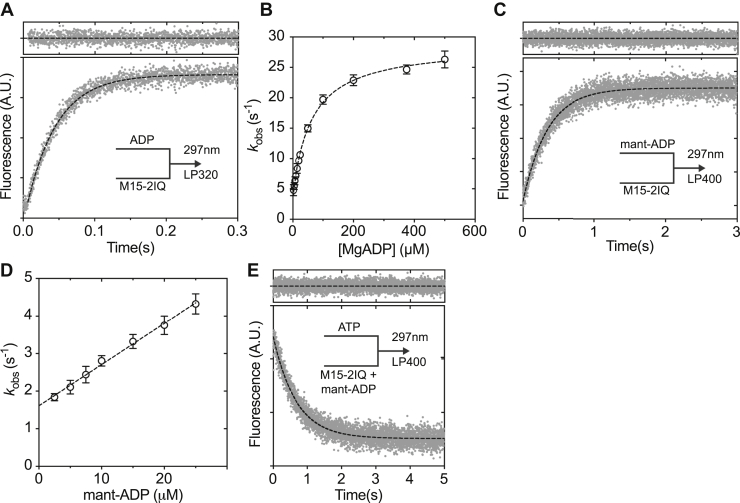
**Kinetics of ADP binding to EGFP-M15-2IQ.***A*, enhancement of intrinsic protein fluorescence as 200-μM MgADP bound to 0.25-μM EGFP-M15-2IQ in the stopped-flow. After an initial lag phase (∼10 ms), the recorded transient follows a single exponential time course with *k*_obs_ = 23.8 ± 0.2 s^−1^ (*dotted line*, fit residuals above). *B*, observed rate constants vary hyperbolically (*dotted line*) with respect to [MgADP], indicating that ADP binding is at least a two-step process. Referring to Equation 3, the data points were fit to *k*_obs_ = *k*_−2D_ + (*K*_1D_·*k*_+2D_·[ADP])/(1 + *K*_1D_·[ADP]), yielding *k*_+2_ = 25.2 ± 0.4 s^−1^, *k*_−2_ = 3.5 ± 0.3 s^−1^, and 1/*K*_1D_ = 60.6 ± 4.7 μM. *C*, fluorescence enhancement after rapid mixing of 10-μM mant-ADP with 0.25-μM EGFP-M15-2IQ in the stopped flow. The mant fluorophore was excited by FRET from vicinal tryptophan residues. The transient followed a monophasic exponential increase, *I*(*t*) = −1.7*e*^−28.8*t*^ + *C* (*dotted line*, fit residuals above). *D*, observed rate constants varied linearly with [mant-ADP]. Linear regression to *k*_obs_ = *k*_+5_ + *k*_−5_ [ADP] yields *k*_−5_ = 0.11 ± 0.01 μM s^−1^ and *k*_+5_ = 1.6 ± 0.07 s^−1^. *E*, displacement reaction where 0.25-μM EGFP-M15-2IQ was pre-equilibrated with 25-μM mant-ADP, before rapid mixing with 5-mM ATP. The transient followed a single exponential decay with *I*(*t*) = 1.9*e*^−1.5*t*^ + *C* (*dotted line*, fit residuals above). Reaction conditions for all experiments: 20-mM Mops (pH 7.0), 100-mM KCl, 5-mM MgCl_2_, 0.1-mM EGTA at 20 °C. Data are representative of n = 3 independent determinations, except (*E*) (n = 2).

We used enhancement of intrinsic protein fluorescence to measure the kinetics of ADP binding to EGFP-M15-2IQ. Although intrinsic protein fluorescence is well established to be sensitive to structural changes induced by ATP binding and hydrolysis, in muscle myosin, it is also responsive to ADP binding (50, 51). We similarly found that mixing of ADP with EGFP-M15-2IQ induced a robust intrinsic fluorescence transient. EGFP-M15-2IQ (0.25 μM) was rapidly mixed in the stopped flow with ADP under pseudo–first-order conditions while monitoring intrinsic fluorescence. ADP in the reaction was titrated across a concentration range of 2.5 μM to 500 μM. At low [ADP], the observed transients followed a monophasic exponential increase; however, at higher [ADP], an initial lag phase became apparent (Fig. 7*A*). We attempted to fit this transient using a double exponential function but could not resolve the fast phase (*k*_obs1_) within an acceptable error. Instead, we fit the observed transients to a single exponential (*k*_obs2_) at lower [ADP] and excluded the short lag phase from the fitting procedure at higher [ADP]. Observed rate constants for the slower phase (*k*_obs2_) varied hyperbolically with respect to [ADP] (Fig. 7*B*), consistent with ADP binding being at least a two-step mechanism. We modeled the reaction as shown in Equation 3, where myosin and ADP initially form a weakly bound state M(ADP) that isomerizes to a strongly bound M∗⋅ADP state with enhanced fluorescence. These states are equivalent to the myosin nucleotide binding pocket in ADP-bound ‘open’ and ‘closed’ conformations, respectively (30). The presence of the initial lag phase indicates that the formation of the open pocket conformation M(ADP) is not in rapid equilibrium (*i.e.*, *k*_−1D_ >> *k*_+2D_).

(3) $$\text{M}+\text{ADP}\overset{k_{+1\text{D}}\left[\text{ADP}\right]}{\underset{k_{-1\text{D}}}{⇌}}\text{M}\left(\text{ADP}\right)\overset{k_{+2\text{D}}}{\underset{k_{-2\text{D}}}{⇌}}{\text{M}}^{\text{∗}}\cdot \text{ADP}$$

An analytical solution to this mechanism is a double exponential function with observed rate constants as defined in Equations 4 and 5 (52). We fitted the slow observed rate constant (*k*_obs2_) from our data to Equation 5 to yield estimates for the dissociation constant 1/*K*_+1D_ = 60.6 ± 4.7 μM and isomerization rate constants *k*_+2D_ = 25.2 ± 0.4 s^−1^ and *k*_−2D_ = 3.5 ± 0.3 s^−1^ (Fig. 7*B*). The apparent association rate constant *K*_1D_⋅*k*_+2D_ = 0.29 ± 0.04 μM^−1^s^−1^ was extracted from the gradient of the second-order response at low [ADP] (Fig. 7*B*). Although the initial binding of ADP (*K*_1D_) to EGFP-M15-2IQ was low affinity (1/*K*_1D_ ∼ 60 μM), the overall apparent affinity is strongly influenced by *K*_2D_, the isomerization equilibrium constant (30). Equation 6 is an expression for the apparent dissociation equilibrium constant (*K*_D_) of a two-step binding mechanism, rearranged from Nyitrai and Geeves (2004) to be consistent with Figure 2 and Equation 3. Equation 6 shows that if the isomerization *K*_2D_ << 1, then the apparent dissociation constant will equal the affinity of the open ADP-bound conformation, *K*_5_ = 1/*K*_1D_. Conversely, if the isomerization *K*_2D_ >> 1, the apparent dissociation constant will be significantly tighter, with *K*_5_ = 1/(*K*_1D_⋅*K*_2D_). We calculated the isomerization equilibrium constant *K*_2D_ using the relation *K*_2D_ = *k*_+2D_/*k*_−2D_ = 7.2 ± 0.6 and substituted into Equation 6 to yield the apparent dissociation constant *K*_5_ = 7.4 ± 0.8 μM.

(4) $$k_{\text{obs}1}\;=\;k_{+1\text{D}}\left[\text{ADP}\right]\;+\;k_{-1\text{D}}$$

(5) $$k_{\text{obs}2}\;=\;k_{-2\text{D}}\;+\;\frac{K_{+1\text{D}}k_{+2\text{D}}\left[\text{ADP}\right]}{1\;+\;K_{+1\text{D}}\left[\text{ADP}\right]}$$

(6) $$K_{D}\;=\;K_{5}\;=\;\frac{1}{K_{1D}\;\cdot \left(K_{2D}\;+\;1\right)}$$

ADP binding to myosin was independently probed using the fluorescent analog, mant-ADP. EGFP-M15-2IQ (0.25 μM) was rapidly mixed under pseudo–first-order conditions in the stopped flow while titrating mant-ADP concentrations. The mant fluorophore was excited at 297 nm using FRET from vicinal tryptophan residues. Fluorescence transients followed a monophasic exponential time course with no lag phase (Fig. 7*C*). Observed rate constants (*k*_obs_) varied linearly with respect to [mant-ADP], and we calculated the apparent association rate constant *k*_−5_ = 0.11 ± 0.01 μM^−1^s^−1^ from the gradient of this second-order response, using the relation *k*_obs_ = *k*_−5_⋅[ADP] + *k*_+5_ (Fig. 7*D*). The apparent dissociation constant *k*_+5_ = 1.6 ± 0.07 s^−1^ was measured from the *y*-axis intercept (Fig. 7*D*). A displacement reaction was used to independently confirm mant-ADP dissociation kinetics. EGFP-M15-2IQ (0.25 μM) preequilibrated with 25-μM mant-ADP (>than *K*_d_ to ensure saturation of the active site) was rapidly mixed with 5-mM ATP in the stopped flow (Fig. 7*E*). The observed fluorescence decay followed a single exponential time course with *k*_obs_ = 1.50 ± 0.02 s^−1^. The dissociation equilibrium constant was calculated using the relationship, *K*_5_ = *k*_+5_/*k*_−5_ = 13.6 ± 1.2 μM, in reasonable agreement with our estimate from intrinsic fluorescence.

**Figure 8:**
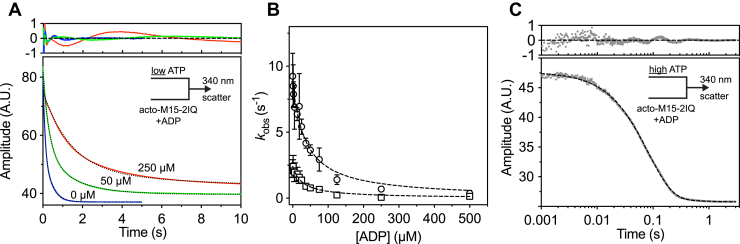
**ADP binding to actomyosin.***A*, the actin-attached dissociation constant (*K*′_5_) of ADP from EGFP-M15-2IQ was measured using a displacement reaction. EGFP-M15-2IQ (0.25 μM), actin (0.5 μM), and ADP (0–0.5 mM) were pre-equilibrated before rapid mixing with ATP (50 μM) in a stopped-flow spectrophotometer. ATP induced dissociation of the actomyosin complex was monitored using orthogonally scattered light at 340 nm. The ATP concentration was chosen to allow ATP and ADP to compete for binding to EGFP-M15-2IQ. Example traces are shown for 0 μM (*blue*), 50 μM (*green*), and 250 μM ADP (*red*) and are shown with biphasic exponential fits overlaid (*dotted line*, residuals above). *B*, dependence of fast (*circles*) and slow (*square*) observed rate constants upon [ADP]. The fast phase was interpreted as direct binding of ATP to actomyosin. Fitting of the fast phase to an inverse-hyperbola reveals half-maximal inhibition at 36.1 ± 5.7 μM ADP. *C*, ADP release from actomyosin was measured using a displacement reaction. EGFP-M15-2IQ (0.25 μM) was pre-equilibrated with actin (0.5 μM) and saturating ADP (0.5 mM), before rapid mixing with 5-mM MgATP in the stopped flow. The high concentration of ATP prevents ADP rebinding. The light scattering transient is shown fit to a double-exponential decay, *I*(*t*) = 21.4*e*^−12.1*t*^ + 0.5*e*^−1.8*t*^ + *C* (*dotted line*, residuals above). Reaction conditions for all experiments: 20-mM Mops (pH 7.0), 100-mM KCl, 5-mM MgCl_2_, 0.1-mM EGTA at 20 °C. Data are representative of n = 3 independent determinations, except (*C*) (n = 2).

We were unable to directly measure the kinetics of ADP binding to actomyosin using either intrinsic fluorescence or mant-labeled ADP analogs, as these transients had poor signal-to-noise. Instead, we indirectly probed the actin-attached ADP dissociation constant (*K*′_5_) by measuring ATP binding to actomyosin in the presence of ADP competing for the active site. We modeled this reaction according to Equation 7, where ADP is in rapid equilibrium with ATP binding to actomyosin (44). EGFP-M15-2IQ (0.125 μM) pre-equilibrated with actin (0.25 μM) and ADP was rapidly mixed in the stopped-flow experiment with 50-μM ATP, while measuring orthogonally scattered light to monitor dissolution of the actomyosin complex. The low concentration of ATP (50 μM, defined as ATP_0_) used in these titrations bound slowly to actomyosin (Fig. 4*B*) and allowed for ADP to compete in rapid equilibrium (*i.e.*, *k*′_−5_⋅[ADP] >> *K*′_1_⋅*k*′_+2_ [ATP_0_]). Experimental transients were fit to a biphasic exponential decay in the absence of ADP (Fig. 8*A*), consistent with our earlier data that actomyosin occupies nucleotide-sensitive and nucleotide-insensitive states (Fig. 4*B*). Fit residuals showed evidence of a systematic deviation, but we could not consistently fit triple exponentials (Fig. 8*A*). Both fast and slow rate constants reduced hyperbolically as ADP was titrated from 0 to 500 μM (Fig. 8*B*). We focused on the observed fast phase (*k*_obs1_) and interpreted this as direct binding of ATP to the nucleotide-sensitive actomyosin fraction. Fast-phase rate constants were fit to Equation 8, where ATP_0_ represents the initial [ATP] that is held constant throughout the titration (44). Fitting to the fast phase yielded the actin-attached ADP dissociation constant, *K*′_5_ = 36.1 ± 5.7 μM. These data indicate a moderate thermodynamic coupling between the MYO15 nucleotide and actin-binding sites (*K*′_5_/*K*_5_ = 4.9), with the affinity of actomyosin for ADP (*K*′_5_) being 4.9-fold lower than the affinity of myosin for ADP (*K*_5_).

(7) $$\text{AM}\cdot \text{ADP}\overset{K_{5}^{\prime }}{⇌}\text{AM}+\text{ADP}+\text{ATP}\overset{K_{+1}^{\prime }k_{+2}^{\prime }}{\to }\text{A}+\text{M}\cdot \text{ATP}$$

(8) $$k_{\text{obs}}=\frac{K{\text{′}}_{1}k{\text{′}}_{+2}\left[{\text{ATP}}_{0}\right]}{1+\frac{\left[\text{ADP}\right]}{K\prime_{5}}}$$

In myosin motors that are specialized for processive motion along cellular actin filaments (*i.e.*, MYO5A, MYO6), the rate-limiting step of the ATPase cycle is actin-attached ADP release (*K*′_5_), ensuring that the motor has a high duty ratio and remains attached to actin (53, 54). To determine if MYO15 has a similar characteristic, we measured actin-attached ADP release using a modification of the ADP competition experiment above. EGFP-M15-2IQ (0.125 μM) was pre-equilibrated with actin (0.25 μM) and ADP (500 μM) to saturate the nucleotide-binding site. The time course of light scattering was monitored after rapid mixing with 5-mM ATP in the stopped flow. ATP binding to actomyosin was now rapid (∼200 s^−1^) and irreversible under these conditions (Fig. 4*B*), and thus, ATP binding was rate-limited by ADP release from the active site (Equation 7). Experimentally observed light scattering transients were well fit to a double exponential transient with observed rate constants *k*_obs1_ = 12.5 ± 0.5 s^−1^ and *k*_obs2_ = 2.1 ± 0.5 s^−1^. The normalized amplitude of *k*_obs1_ was ∼97%, and we interpreted this to represent ADP release from actomyosin (*K*′_5_). Unlike MYO5A and MYO6 where the ATPase is rate-limited by actin-attached ADP release (53, 54), our data show that actin-attached ADP release (∼12 s^−1^) is not slow enough to solely rate-limit the MYO15 ATPase cycle (*k*_cat_ = 6 s^−1^).

## Discussion

### Client specificity of UNC45 chaperones for the MYO15 motor domain

This study expands upon our previous work that the striated-muscle UNC45B co-chaperone is necessary to promote folding of the MYO15 motor domain (16). In the previous study, we showed that expression of motor domain in *Sf*9 cells resulted in aggregated, inactive protein unless UNC45B was coexpressed (16). In the present study, we show that the more ubiquitously expressed UNC45A can similarly promote folding of the MYO15 motor domain in *Sf*9 insect cells and is significantly more efficient than UNC45B. UNC45A and UNC45B are co-chaperones that activate HSP90-dependent folding of the conventional muscle myosin motor domain (34, 35) and also fold several unconventional myosin motor domains, including classes XIV and XV (16, 36). Our experiments also show that the MYO15 motor domain purified from *Sf*9 cells coexpressing either UNC45A or UNC45B exhibit similar steady-state activities (*k*_cat_ and *K*_ATPase_), suggesting that the motor domains were functionally similar. These data do not support the hypothesis that different UNC45 chaperones post-translationally regulate motor domain activity, although we cannot rule out more subtle differences in ATPase activity that would be overlooked by steady-state assays. These experimental results used *Sf*9 cells infected with a dual-promoter baculovirus expressing either mouse UNC45A or UNC45B, in conjunction with HSP90AA1. We further replicated these findings using *Sf*9 cells engineered to stably express only mouse UNC45A, or UNC45B, demonstrating that endogenous insect HSP90 orthologs were sufficient to function with UNC45. Our findings confirm a recent report where overexpression of the *C. elegans* UNC45 ortholog was sufficient to fold muscle myosin (MHC-B) in *Sf*9 cells without the overexpression of HSP90 (55). The *Sf*9–UNC45 cell lines developed here facilitate the expression of recombinant MYO15 and may prove helpful for other proteins and myosin motors that require UNC45 paralogs to fold and mature correctly.

Cochlear hair cells express both UNC45A and UNC45B (https://umgear.org), and we hypothesize that either one of these chaperones is required for the correct folding of MYO15 *in vivo*. Intriguingly, loss-of-function mutations in *UNC45A* are associated with a human form of syndromic deafness (56), although it is unknown whether this is caused by defective hair cell stereocilia formation, similar to the phenotype caused by pathogenic *Myo15* variants in mouse (3, 5). Why might hair cells express both UNC45 paralogs? We suspect that the answer lies with UNC45 substrate specificity. HSP90-dependent folding of conventional smooth muscle myosin can be catalyzed *in vitro* by either UNC45 paralog, yet UNC45A displays increased folding efficiency toward this substrate (35). Furthermore, while there appears to be some degree of functional overlap *in vitro*, developmental studies in zebrafish (*Danio rerio*) show that *unc45a* and *unc45b* are not functionally redundant *in vivo* (57). Our results suggest a similar substrate selectivity for MYO15, with UNC45A being significantly more efficient at folding the motor domain than UNC45B. The expression of both paralogs in hair cells may therefore indicate UNC45 isoforms engaging with a broader spectrum of client proteins. A number of myosin proteins are critical for hearing, including MYO3A/B (58, 59, 60), MYO6 (61), MYO7A (62), and MYO15 (5). MYO3A, MYO6, and MYO7A have all been successfully purified from *Sf*9 cells without the use of UNC45 coexpression, suggesting that this chaperone is not a prerequisite for folding (63, 64, 65, 66). It is interesting to note that the UNC45 family chaperones also target the unfolded myosin motor domain *in vitro* (34, 55, 67) and potentially do so in response to tissue trauma *in vivo* (68). An exciting hypothesis is that UNC45 paralogs may maintain hair cell proteostasis and refold myosin motors in stereocilia as they are denatured and damaged. Given that mammalian hair cells do not regenerate and must survive throughout the animal's lifetime, we speculate this type of myosin quality control may be critical for long-lived sensory function.

### The ATPase mechanism of MYO15

Our experiments identify the key motor domain characteristics of MYO15 to be (1) actin-attached ADP release as the slowest overall reaction step, (2) a moderate affinity for ADP with weak thermodynamic coupling between nucleotide and actin binding, (3) rapid ATP hydrolysis with an equilibrium constant (*K*_3_) close to unity, (4) weak binding of ATP, and (5) a moderate affinity for actin in the absence of nucleotide, compared with that of most myosins. Thermodynamic and kinetic rate constants from our steady-state and pre–steady-state transient analyses are summarized in Tables 1 and 2.

**Table 2:** Summary of key ATPase kinetic parameters

Parameter	Value
ATP binding	
*K*_1_⋅*k*_+2_ (μM^−1^ s^−1^)	0.17 ± 0.01 _intrinsic_ 0.15 ± 0.002 _mantATP_
1/*K*_1_ (μM)	1898 ± 145 _intrinsic_
*k*_+2_ (s^−1^)	322 ± 10 _intrinsic_
*K*′_1_⋅*k*′_+2_ (μM^−1^ s^−1^)	0.12 ± 0.02 _light scatter_ 0.12 ± 0.01 _mantATP_
1/*K*′_1_ (μM)	2749 ± 268 _light scatter_
*k*′_+2_ (s^−1^)	307 ± 15 _light scatter_
ADP binding	
*k*_+5_ (s^−1^) ^app^	2.1 ± 0.4 (= *K*_D_·*k*_−5_) _intrinsic_ 1.5 ± 0.02 s^−1^_mant ADP_
*k*_−5_ (μM^−1^⋅s^−1^) ^app^	0.29 ± 0.04 _intrinsic_ 0.11 ± 0.01 _mant ADP_
*K*_5_ = *K*_D_ (μM) ^app^	7.4 ± 0.8 _intrinsic_ 13.6 ± 1.2 _mant ADP_
*k*′_+5_ (s^−1^) ^app^	12.5 ± 0.5 _light scatter_
*k*′_−5_ (μM^−1^ s^−1^) ^app^	0.35 ± 0.06 (= *k*′_+5_/*K*′_5_) _light scatter_
*K*′_5_ = *K*_AD_ (μM) ^app^	36.1 ± 5.7 μM _light scatter_
*K*_AD_/*K*_D_	4.9 (= *K*′_5_/*K*_5_^_intrinsic_^) 2.7 (= *K*′_5_/*K*_5 mant ADP_)
Hydrolysis and phosphate release	
*k*_+3_ + *k*_−3_ (s^−1^)	>50.2 ± 5.7_quenched flow_
*K*_3_	1.34 ± 0.07 _quenched flow_
*k*_+4_ (s^−1^)	0.08 ± 0.001 _MDCC-PbP_
*K*_9_·*k*′_+4_ (μM^−1^ s^−1^)	0.13 ± 0.004 _MDCC-PbP_
Actin binding	
*k*_+6_ (μM^−1^ s^−1^)	3.18 ± 0.15 _pyrene_
*k*_−6_ (s^−1^)	0.65 ± 0.3 _pyrene_^a^
1/*K*_6_ = *K*_A_ (nM)	200 ± 100 _pyrene_
*k*_+10_ (μM^−1^ s^−1^)	1.75 ± 0.09 _pyrene_
*k*_−10_ (s^−1^)	0.55 ± 0.17_pyrene_
1/*K*_10_ = *K*_DA_ (nM)	310 ± 97 _pyrene_
*K*_DA_/*K*_A_	1.6
Other	
*K*′_11_	3.8 ± 1.0 _light scatter_
*k*′_+11_ (s^−1^)	30.9 ± 1.9 s^−1^_light scatter_
*k*′_−11_ (s^−1^)	8.1 ± 2.2 (= *k*′_+11_/*K*′_11_)

Assay conditions: 20-mM Mops, pH 7.0, 100-mM KCl, 5-mM MgCl_2_, 0.1-mM EGTA at 20 ± 0.1 °C.

^app^ apparent values.

a Inferred from *y*-axis intercept.

The basal ATPase rate of the MYO15 motor domain in the absence of actin (see Table 1) was rate-limited by phosphate release (*k*_+4_), with the addition of actin stimulating the steady-state activity ∼77-fold to a maximal turnover rate (*k*_cat_) of ∼6 s^−1^ at 20 °C. Our previous work showed that *k*_cat_ was insensitive to salt concentrations between 10- and 100-mM KCl (16), and our measurement of *k*_cat_ using 10-mM KCl (this study) was in good agreement with these previous estimates. We were unable to identify a single transition that solely rate-limited the catalytic cycle. Our transient kinetic analysis showed that ATP binding to actomyosin (*K*′_1_⋅*k*′_+2_) was >125 s^−1^ at 2-mM MgATP and thus too fast to be rate-limiting at steady state (Fig. 4*B*). Actin-detached hydrolysis (*k*_+3_ + *k*_−3_) was >50 s^−1^ measured using quenched flow at 0.5-mM ATP and was similarly too fast to be rate-limiting (Fig. 5*A*). We were unable to probe the maximum rate of actin-activated phosphate release (*k*′_+4_) because of the weak affinity of M⋅ADP⋅P_i_ for actin (*K*_9_) at the physiological salt concentrations (100-mM KCl) used in our study. Despite this, the second-order response was linear up to ∼6 s^−1^ at 50-μM actin with no sign of deviation (Fig. 5*C*), indicating that phosphate release was not close to saturation, and unlikely to be rate-limiting either. ADP release from actomyosin was the slowest measured transition at ∼12 s^−1^, and our study suggests it is a major contributor toward rate-limiting the MYO15 ATPase cycle.

Both myosin and actomyosin had moderate affinities for ADP in the overall range of 7 to 36 μM, and thermodynamic coupling between nucleotide and actin binding was weak (*K*_AD_/*K*_D_ = 2.7–4.9, *K*_DA_/*K*_A_ = 1.6, see Table 2). This indicates that actin binding to MYO15 does not significantly lower its affinity for ADP and is a key enzymatic adaptation that allows for a load bearing, strongly bound actomyosin interaction to form under intracellular ADP concentrations (∼50 μM) (69). This is in contrast to motors with high thermodynamic coupling ratios (*K*_AD_/*K*_D_ > 30), such as fast skeletal myosin (70), where actin binding leads to a rapid release of ADP from the active site. Our data show that ADP binding and release from MYO15 involved at least a two-step mechanism. We observed both a lag phase and saturation of observed rate constants as ADP bound to MYO15 (Fig. 7, *A* and *B*). These data are consistent with a two-step sequential binding mechanism and suggest that MYO15 undergoes an ADP-bound structural isomerization. Two-step ADP binding is proposed as a common mechanism for all hydrolysis-competent myosin motors, where ADP binds the nucleotide pocket in an ‘open’ configuration, before isomerization to an ADP-bound ‘closed’ configuration (30, 31). When strongly bound to actin, a critical parameter determining myosin function is the rate of isomerization, from the ADP-bound closed, to open isomer (also referred to as *K*_CO_). This isomerization can be opposed by mechanical load exerted on the actomyosin cross bridge, and modulate the rate of ADP release and thus the lifetime of the strongly bound actomyosin state (71, 72, 73, 74).

We were unable to experimentally probe the kinetics of ADP-bound isomerization for actomyosin, as neither ADP nor mant-ADP generated robust fluorescence transients upon binding. Instead, we measured ADP release from actomyosin using a displacement reaction and observed a predominantly monophasic exponential decay of ∼12 s^−1^ (Fig. 8*C*). A monophasic transient might be expected from a two-step mechanism if the nucleotide-binding pocket is biased toward the open ADP-bound configuration because the closed ADP-bound species are unable to populate in the experimental premixtures. If this were the case, our displacement reaction measured ADP release primarily from the open isomer, and it follows that any ADP-bound isomerization will be kinetically invisible in our experiment. ADP release from the closed isomer may only be kinetically accessible approaching from the hydrolysis and phosphate release states, similar to muscle myosin (75). In further support of an actin-attached ADP isomerization, we observed that ATP binding to nucleotide-free actomyosin was biphasic, with both phases saturating with increasing [ATP] (Fig. 4*B*). This behavior has been observed in class I myosin and is consistent with the nucleotide-binding pocket of actomyosin isomerizing between open and closed states (41, 42).

Similar to the other myosin tail homology 4–4.1, ezrin, radixin, moesin myosins (MYO7A, MYO10), quenched-flow experiments show that the MYO15 motor domain has a hydrolysis equilibrium constant (*K*_3_) near unity, indicating that M⋅ATP and M⋅ADP⋅P_i_ states are equally populated at the steady state (47, 48). This enzymatic adaptation may allow for a parallel flux through the actin-attached hydrolysis (*i.e.*, M⋅ATP > AM⋅ATP > AM⋅ADP⋅P_i_) pathway, in addition to actin-detached hydrolysis (*i.e.*, M⋅ATP > M⋅ADP⋅Pi > AM⋅ADP⋅P_i_). The extent of this contribution depends upon the flux of actin-attached hydrolysis (*k*′_+3_ + *k*′_−3_) and the affinity of M⋅ATP for actin (*K*_8_, see Fig. 2). The latter is challenging to measure experimentally and has been previously estimated from global numerical simulations of the ATPase cycle (47, 48). In the case of MYO7A and MYO10, the effect of the actin-attached pathway is to divert enzymatic flux through a slower hydrolysis pathway and contribute to rate-limiting the ATPase cycle at higher actin concentrations (47, 48). It remains unclear from our study the extent of this contribution, if any, to the MYO15 ATPase cycle.

ATP binding to the MYO15 motor domain was notably weak with the rate constant not fully saturated at 5-mM ATP (Figs. 3*B* and 4*B*). This arises from the initial binding step having a high dissociation constant (1/*K*′_1_ = 2.7 mM) and not from the subsequent isomerization step (*k*′_+2_) that was rapid (∼300 s^−1^) and irreversible. The weak binding of ATP suggests there may be steric hindrance either in the nucleotide-binding pocket or gaining access to it. MYO6 exhibits even weaker ATP binding than MYO15 (1/*K*′_1_ > 14 mM), and this has been attributed to a unique insert 1 in the nucleotide-binding pocket that allows ADP to bind preferentially over ATP (54, 76). MYO15 does not have an equivalent insert; however, the motor domain structure has not been reported and it may reveal other steric effects in the binding pocket to explain this behavior. A possibility is that variations in loop 1 may contribute to the weak binding of ATP. MYO15 has an alternatively spliced cassette micro–exon 8 that inserts 2 amino acids (‘IK’) into loop one (2), and this may interfere with access to the pocket or propagate changes to the nucleotide-binding site *via* altered flexibility (77, 78). MYO15 purified for this study contains the micro–exon 8–encoded insertion ‘IK,’ and it will be interesting to characterize the motor domain lacking these two residues.

The affinity of MYO15 for actin, either ADP bound (*K*_10_) or nucleotide free (*K*_6_), was relatively low, in the range of several hundred nanomolars, compared with the high affinity of MYO5A/MYO6, for example (53, 54). The lower affinity results from a significant off-rate from actin (*k*_−6_, *k*_−10_). While this estimate has a larger uncertainty due to the extrapolation of the off-rate from the *y*-axis intercept (Fig. 6*B*), it does suggest a comparable actin affinity with MYO10, which is closely related phylogenetically (47, 79, 80). MYO10 exhibits a small fractional quench of pyrene-labeled actin and is proposed to engage a subtly different actin-binding interface from other myosins (47). Cryo-EM reconstructions of the MYO10 motor domain at 9-Å resolution have yet to reveal a structural basis for this (81). The different actin-binding topology may relate to MYO10 being kinetically optimized to move along bundles of actin filaments, in preference to individual filaments (81, 82, 83). We expect MYO15 to also be specialized for trafficking along bundles of actin filaments, given that stereocilia actin filaments are extensively cross-linked with FSCN1/2, PLS1, ESPN, and TRIOBP (84, 85, 86). If this is true, we may expect some kinetic parameters for MYO15 to be altered when measured on bundled actin *versus* single actin filaments, similar to MYO10 (81).

### Duty ratio of MYO15

The duty ratio of a myosin molecular motor is defined as the fraction of its mechanochemical cycle spent attached to the actin filament (87). Under saturating ATP, the AM (apo) state is not significantly occupied at steady state, and thus, the duration spent attached to actin is dominated by the strongly bound AM⋅ADP state. The duty ratio can be expressed as follows (Equation 9).

(9) $$\text{duty ratio}=\frac{k_{cat}}{k_{-AD}}$$

Using our measured values for *k*_cat_ and *k*_−AD_ (*k*′_+5_), we estimate that MYO15 has a duty ratio = 0.48 indicating that the motor domain spends ∼48% of its cycle strongly bound to an actin filament. This calculation assumes that entry to the AM⋅ADP state is not rate-limited by ATP binding (*k*′_+2_), ATP hydrolysis (*K*_3_), or actin-attached phosphate release (*K*_9_⋅*k*′_+4_) and omits the dependence of the duty ratio upon [actin]. The duty ratio calculated above would be achieved at a hypothetical infinite [actin], and we speculate this may be biologically relevant considering that MYO15 operates within the densely packed actin filaments of the stereocilia actin core. The duty ratio calculated here differs from our previous estimate of >0.9 that was inferred from the dwell time of actin-attached binding events in an optical trap (16). Uncertainties in the two-step ADP release mechanism from actomyosin may account for this difference; an ADP isomerization step (*K*_CO_) slower than 12 s^−1^ would increase the steady-state population of myosin in strongly actin-bound states, and thus the duty ratio. Furthermore, if ADP release from actomyosin is force sensitive, such as shown for MYO1B (74), then the low pico-Newton (pN) forces experienced in the optical trap may retard ADP release and further increase the duty ratio. Future experiments using force-feedback optical trapping are needed to test if ADP release from MYO15 is load dependent and if so whether a dimerized molecule could gate ADP release to further increase overall processivity, similar to MYO5A (71, 72). For these reasons, we consider the duty-ratio estimate of 0.48 in our present study to be a lower bound.

### Implications for MYO15 function within hair cells

The cellular functions of myosin motors have been classified into four broad overlapping categories, based upon the kinetic signature of their motor domains (29, 30, 31). This scheme uses four motor domain parameters: (1) duty ratio, (2) thermodynamic coupling of actin and nucleotide-binding (*K*_AD_/*K*_D_), 3) load dependence of ADP release, and (4) *K*_C/O_, the equilibrium constant of actin-attached ADP isomerization (from closed to open nucleotide pocket). The kinetic adaptations revealed in our present study show that MYO15 has an intermediate duty ratio (∼0.5) in addition to low thermodynamic coupling between nucleotide- and actin-binding sites. Combined, these characteristics enable the MYO15 motor domain to spend at least 50% of its mechanochemical cycle attached to actin and to simultaneously bind actin and ADP allowing for a force-bearing cross bridge to form. These characteristics are consistent with a monomeric MYO15 molecule behaving as a strain sensor, where force applied to the motor domain can modulate ADP release and its actin-attached lifetime. If MYO15 molecules were to oligomerize into an ensemble, these same characteristics could also give rise to kinetic gating between motor domains to allow for longer-distance processive movement along an actin filament. Although our data are suggestive of strain sensitivity, the potential for this behavior needs to now be directly tested using a technique such as force feedback optical trapping (74, 88).

There is significant evidence that MYO15 molecules can move processively *in vivo*. MYO15 isoforms accumulate at the distal tip of hair cell stereocilia (4, 8), and time-lapse studies show a continual flux of motors toward the stereocilia tip (89). Furthermore, EGFP-tagged MYO15-2 (Fig. 1*A*, isoform 2) traffics toward the distal tips of actin-based filopodia in COS-7 cells (4), in a striking parallel with MYO10 (18). MYO15-2 does not appear to move anterogradely in large puncta along filopodia (17), suggesting that they instead move in small packets consisting of a few molecules, similar to MYO10 (19). Consistent with this, small packets of MYO15 are observed in-transit along the stereocilia shaft in paraformaldehyde-fixed hair cells (7). With an intermediate duty ratio (∼0.5), our kinetic study predicts that MYO15 needs to oligomerize to move processively within stereocilia. How this might be achieved is unclear. While MYO15 lacks any coiled-coil motifs, it may use a cargo-mediated mechanism similar to MYO6 and MYO7A, where accessory proteins activate motility by driving oligomerization of the myosin heavy chain (20, 90, 91). MYO15 binds at least four proteins in hair cells, including WHRN, EPS8, guanine nucleotide-binding protein G_i_ subunit alpha, and G-protein signaling modulator 2. These proteins are trafficked as a complex by MYO15 and regulate stereocilia elongation (17, 23, 24, 27, 28). We speculate that an additional function of these proteins, or a hitherto unidentified partner, is to oligomerize MYO15 and to activate processive motility toward the stereocilia tip.

In addition to trafficking within stereocilia, MYO15 concentrates at the stereocilia tip where active MET channels are located (92). Recent studies have shown that the MYO15–WHRN complex binds *via* CIB2 (93, 94) to TMC1 and TMC2, pore-forming subunits of the MET channel (95, 96). Our finding that the MYO15 motor domain may exhibit strain sensitivity, although requiring further confirmation, does suggest that MYO15 could also act as a force-sensitive element bridging the membrane and actin cytoskeleton at the stereocilia tip. While the physiological function of this remains speculative, MYO15 is ideally placed to respond to tension exerted during auditory mechanotransduction. Understanding how MYO15 oligomerizes *in vivo* is a key question for future experiments, with the monomer to oligomer transition potentially controlling the switch between force-sensing and processive activity.

Our kinetic study represents an important step toward understanding how pathogenic mutations in *MYO15A* cause human deafness DFNB3. More than 300 mutant alleles of *MYO15A* have been identified that cause DFNB3, and a significant percentage of these are missense mutations substituting residues in the motor domain (15). The effects of these mutations are unknown. Our detailed study provides a thermodynamic and kinetic “fingerprint” of the wild-type motor domain, and will allow the precise effects of deafness causing mutations to be ascertained. Our results will also help reveal the activity of full-length MYO15 isoforms that have distinct cellular functions in the assembly and maintenance of the stereocilia actin core (8, 15). MYO15 isoforms are distinguished by a N-terminal domain that precedes the motor domain (Fig. 1*A*). The N-terminal domain of MYO15-1 is notable for both its size (∼1200 aa) and unusually high proline content (17%). In class I myosins, the artificial switching of N-terminal domains between the MYO1B and MYO1C motor domain enables strain-sensitive binding to actin filaments (74, 97, 98, 99). We hypothesize that the N-terminal domain may similarly regulate the motor activity of MYO15-1 and endow this molecule with unique properties. By comparison with the motor domain activity reported in our present study, we hope to understand how the enzymatic activities of full-length MYO15 isoforms differ and ultimately how they contribute to the stereocilia development and maintenance processes essential for lifelong hearing.

## Experimental procedures

### General reagents

Reagents were of the highest grade available from MilliporeSigma, unless otherwise stated. ATP and ADP nucleotides were prepared as equimolar stocks with magnesium acetate at pH 7.0 and quantified by absorbance at 259 nm (ε = 15,400 M^−1^ cm^−1^). mantATP-labeled ATP and ADP stocks (BIOLOG) were quantified by absorbance at 255 nm (ε = 23,300 M^−1^ cm^−1^). MDCC-PbP (45) was the generous gift of Dr Howard White (University of Virginia Medical School). ATP [gamma-32P] was from Perkin Elmer.

### Actin purification and labeling

Rabbit skeletal actin was extracted from muscle acetone powder and labeled on Cys-374 using *N*-(1-pyrene)-iodoacetamide (Thermo Fisher Scientific) as needed (100, 101). Actin for steady-state ATPase measurements was purified through two rounds of polymerization/depolymerization with ultracentrifugation. The concentration of polymerized actin was determined by measuring the absorbance at 290 nm (ε = 26,600 M^−1^ cm^−1^) and dialyzed extensively against 4-mM Mops (pH 7.0), 1-mM MgCl_2_, 0.1-mM EGTA, 1-mM DTT, 1-mM NaN_3_ before use. All actin used for stopped-flow spectrophotometry (unlabeled and pyrene-conjugated) were purified by size-exclusion chromatography (16/60 Sephacryl S-300 HR, Purifier 10, GE Healthcare) with isocratic elution in G-buffer (2-mM Tris HCl (pH 8.0), 0.2-mM ATP, 0.1-mM CaCl_2_, 1-mM DTT), before polymerization and extensive dialysis against 20-mM Mops (pH 7.0), 100-mM KCl, 5-mM MgCl_2_, 0.1-mM EGTA, and 1-mM DTT to remove contaminating nucleotides. Actin concentrations were determined at 290 nm (ε = 26,600 M^−1^ cm^−1^) and additionally at 344 nm (ε = 22,000 M^−1^ cm^−1^) for pyrene-labeled stocks. A correction factor was applied for pyrene actin, A_corr_ = A_290_ – 0.127 ∗ A_344_. Pyrene labeling of actin was typically 80 to 90% (mol: mol). All actin filaments used for stopped-flow experiments were stabilized with a molar equivalent of phalloidin (Sigma).

### Sf9–UNC45 stable cell generation and Western blotting

Transient expression of untagged UNC45A or UNC45B in *Sf*9 cells was driven using the opIE2 promoter in *pIB* (Thermo Fisher Scientific). Expression of *pIB* confers resistance to the antibiotic blasticidin allowing for positive selection in *Sf*9 insect cells. The *pIB* empty vector was linearized with EcoRI and XhoI before agarose electrophoresis and gel purification (NucleoSpin, Takara Bio). The ORFs encoding mouse UNC45A and mouse UNC45B were PCR-amplified from *pFastbac Dual Unc45(a/b)/Hsp90aa1* (see below) using PrimeSTAR HS (Takara Bio, CA) and primers as shown (Table 3). A stop codon was introduced into the reverse primers to prevent expression of the V5-His_6_ tag within the *pIB* vector backbone. Amplicons were ligated into linearized *pIB* using In-Fusion HD EcoDry (Takara Bio) and transformed into *Escherichia coli* bacteria (Stellar, Takara Bio) following the manufacturer's protocol. Recombinant clones were screened by restriction digest with EcoRI and XhoI, and correctly structured plasmids were Sanger-sequenced to confirm the ORF.

**Table 3:** DNA oligonucleotides used in this study

Primer	Sequence
pFastbac Dual.Unc45a-Forward	5′-CGA CGA GCT CAC TAG TGC CAC CAT GAC TGT GAG TGG CCC GGA GAC C-3′
pFastbac Dual.Unc45a-Reverse	5′-CAA GCT TGT CGA GAC TGC AGT CAC TCT CCG TCC TGG TTG GGT TG-3′
pIB.Unc45a-Forward	5′-CAG TGT GGT GGA ATT CGA AAC CAT GAC TGT GAG TGG CCC GGA GAC C-3′
pIB.Unc45a-Reverse	5′-GCC CTC TAG ACT CGA GTC ACT CTC CGT CCT GGT TGG GTT G-3′
pIB.Unc45b-Forward	5′-CAG TGT GGT GGA ATT CGA AAC CAT GGC AGA GGC TGA AGC GGC ACA G-3′
pIB.Unc45b-Reverse	5′-GCC CTC TAG ACT CGA GCT ATG ACA CCG GCT TGA TGA AGC C-3′
pIB.Genomic-Forward	5′-CCC TTC CGG CTG GCT GGT TTA-3′
pIB.Genomic-Reverse	5′-TGT CGG GTT TCG CCA CCT CTG-3′

*Sf*9 cells were maintained at 27 °C in HyClone SFX (GE Healthcare) and transfected in suspension culture using endotoxin-free *pIB-Unc45a* or *pIB-Unc45b* plasmid DNA (NucleoBond Maxi EF, Takara Bio). Plasmid DNA was complexed with polyethylenimine (PEI, #24765-1, Polysciences Inc) at a 12:1 (w/w) PEI:DNA ratio and added to *Sf*9 cells in the suspension culture. After 96 h in culture, cells were sparsely seeded into 10-cm culture dishes and media supplemented with 15 μg/ml blasticidin S (Thermo Fisher Scientific) to drive positive selection of *Sf*9 cells with stable integration of the *pIB-Unc45a* or *pIB-Unc45b* plasmid. After approximately 2 weeks in culture, adherent colonies were isolated using glass cloning cylinders (Sigma), transferred to a 96-well plate, and sequentially scaled up to a six-well plate. The identity of each clone was analyzed by genomic PCR and Western blotting. The *pIB* gene expression cassette containing *Unc45a* or *Unc45b* was PCR amplified from *Sf*9 genomic DNA using Ex Taq (Takara Bio) and primers as shown (Table 3). Amplicons of the expected size (∼7.5 kb) were gel-purified and Sanger-sequenced to confirm integrity of the UNC45 transgene. Soluble protein was extracted from *Sf*9–UNC45A and *Sf*9–UNC45B cells using lysis buffer (see below, Expression and purification of the MYO15 motor domain), analyzed by SDS-PAGE (4–20% TGX, Bio-Rad) and blotted onto a polyvinylidene difluoride membrane using semi-dry transfer (Trans-Blot, Bio-Rad). Blots were probed with primary antibodies, mouse IgG anti-UNC45A (SAB1400633, MilliporeSigma), or mouse IgG anti-UNC45B (ab77062), followed by secondary detection with horseradish peroxidase–conjugated goat anti-mouse IgG (AP308P, MilliporeSigma). Chemiluminescence was captured using a charge-coupled camera (ChemiDoc MP, Bio-Rad).

### Baculoviral transfer vector cloning and baculovirus generation

A dual-promoter baculoviral vector (*pFastbac Dual*, Life Technologies) was used to engineer baculovirus expressing either mouse UNC45A (GenBank: AAH04717.1) or UNC45B (GenBank: AAH84585.1) from the polyhedrin promoter, in addition to mouse HSP90AA1 (GenBank: AAH46614.1) from the p10 promoter. UNC45A-, UNC45B-, and HSP90AA1-expressed proteins were not epitope-tagged. The generation of *pFastbac Dual Unc45b*/*Hsp90aa1* was previously described (16). To generate *pFastbac Dual Unc45a*/*Hsp90aa1*, the ORF of mouse *Unc45a* was PCR-amplified from a P8.5 mouse embryo cDNA (Takara Bio) using PrimeStar HS (Takara Bio) and primers as shown (Table 3). Amplicons were ligated into SpeI/PstI linearized *pFastbac Dual Hsp90aa1* (16) using InFusion HD EcoDry (Takara Bio). Correctly recombined clones were isolated and confirmed by Sanger sequencing of both *Unc45a* and *Hsp90aa1* ORFs. *pFastbac1 EGFP-M15-2IQ* and *pFastbac M15-2IQ-EGFP* encoding the truncated mouse MYO15 motor domain (NP_874357.2, aa 1–743) with two LCBDs (IQ domains) and a C-terminal FLAG (DYKDDDK) epitope have been previously described (16). The expressed proteins were (His)_6_-tev-EGFP-M15-2IQ-FLAG (119 kDa) and M15-2IQ-EGFP-FLAG (114 kDa).

Baculoviral transfer vectors were transformed into *E. coli* DH10-Bac cells (Life Technologies), and recombinant bacmid DNA was purified according to the manufacturer's protocol. Recombinant bacmid DNA was transfected into *Sf*9 cells using PEI, as described above, to generate first passage (P1) baculoviral stocks. A dual-promoter baculovirus encoding bovine smooth muscle essential (MLC17B, MYL6) and chicken RLC (MLC20, MYL12B) was previously described (102). First passage baculoviral stocks were amplified in *Sf*9 cells, using a low multiplicity of infection (MOI = 0.1), to generate second passage (P2) virus for expression. All baculoviral stocks were titered using an end-point dilution assay in combination with the *Sf*9-ET (Easy-Titer) reporter cell line (103).

### Expression and purification of the MYO15 motor domain

*Sf*9 cells were maintained in either HyClone SFX (GE Healthcare) or ESF-921 (Expression Systems) at 27 °C in suspension culture. For protein expression, 1 L of *Sf*9 cells were seeded at 2 × 10^6^ cells⋅mL^−1^ and infected simultaneously (MOI = 5) with three baculoviruses encoding EGFP-M15-2IQ, UNC45(A/B)/HSP90AA1, and light chains (ELC/RLC). Alternatively, *Sf*9–UNC45B or *Sf*9–UNC45B cells were infected simultaneously (MOI = 5) with baculoviruses encoding M15-2IQ-EGFP and light chains (ELC/RLC). Infected cells were harvested after 48 h and flash-frozen in liquid nitrogen.

Purification of the motor domain was performed as described previously (16). Briefly, *Sf*9 cell pellets were thawed in 10-mM Mops (pH 7.2), 0.5 M NaCl, 1-mM EGTA, 10-mM MgCl_2_, 2-mM ATP, 0.1-mM DTT, 0.2-mM PMSF, 1-mM NaN_3_, 2 μg mL^−1^ leupeptin, and a protease inhibitor cocktail (Halt EDTA-free; Thermo Fisher Scientific) and lysed using a Dounce homogenizer chilled on ice. *Sf*9 cells lysates were sedimented at 48 kG × 30 min and the supernatant incubated for 3 h on ice with FLAG-M2 affinity gel (Sigma-Aldrich). FLAG-M2 affinity resin was packed into a gravity-flow column and washed with high-salt buffer, 10-mM Mops (pH 7.2), 0.5 M NaCl, 1-mM EGTA, 5-mM MgCl_2_, 1-mM ATP, 1-mM NaN_3_, 0.1-mM DTT, 0.1-mM PMSF, and 1 μg mL^−1^ leupeptin, followed by a low-salt buffer, 10-mM Mops (pH 7.0), 0.1 M NaCl, 1-mM EGTA, 1-mM NaN_3_, 0.1-mM DTT, 0.1-mM PMSF, and 1 μg mL^−1^ leupeptin. The motor domain protein was eluted from the FLAG affinity matrix using a low-salt buffer supplemented with 0.2 mg mL^−1^ 3X FLAG peptide (American Peptide). Eluted motor domain was bound to a strong anion exchanger (5/50 Mono Q GL; GE Healthcare) using a Purifier 10 (GE Healthcare) chromatography system at 4 °C. Bound protein was washed using 5 column volumes of 10-mM Mops (pH 7.0), 0.1 M NaCl, 1-mM EGTA, 0.1-mM PMSF, and 1-mM DTT and eluted with a 160 column-volume gradient to 1 M NaCl. Motor domain fractions eluting at ∼31 mS⋅cm^−1^ were concentrated (10,000 MWCO; Amicon, Millipore) and further purified by size-exclusion chromatography. The motor domain was loaded onto a HiLoad 16/60 column packed with Superdex 200 (GE Healthcare) and purified using isocratic elution with 10-mM Mops (pH 7.0), 250-mM KCl, 0.1-mM EGTA, 1-mM NaN_3_, 0.1-mM PMSF, 1-mM DTT, and 1 μg mL^−1^ leupeptin. The motor domain bound to ELC and RLC eluted as a single peak. The concentration of the final protein complex was determined at 280 nm using the following extinction coefficients, and assuming a 1:1:1 stoichiometry (EGFP-M15-2IQ: ELC:RLC, ε = 93,980 M^−1^ cm^−1^; M15-2IQ-EGFP:ELC:RLC, ε = 88,020 M^−1^ cm^−1^).

To compare the relative folding efficiency of UNC45 isoforms, equal volumes (200 mLs) of *Sf*9 cells were seeded at 2 × 10^6^ cells⋅mL^−1^ and infected with baculovirus simultaneously (MOI = 5) encoding either (A) EGFP-M15-2IQ and ELC/RLC or (B) EGFP-M15-2IQ, UNC45A/HSP90AA1, and ELC/RLC, or (C) EGFP-M15-2IQ, UNC45B/HSP90AA1, and ELC/RLC. After 48 h, protein was isolated as described above. After sedimentation at 48 × kG, small quantities of the total lysate (T) and supernatant (S) were analyzed by SDS-PAGE and chemiluminescent Western blotting with primary antibodies, mouse IgG anti-FLAG (F1804, MilliporeSigma), and mouse IgG anti–alpha-tubulin (T9026, MilliporeSigma). Western blot bands were quantified using automated densitometry (Image Lab, Bio-Rad). The FLAG signal for total (T) and supernatant (S) were first ratioed to their matched alpha-tubulin loading controls, before the (T/S) ratio was calculated. The remaining supernatants from each expression condition were then purified using FLAG affinity, and ion-exchange chromatography, as described above. Absorbance chromatograms at 280 nm were integrated to measure the area under the curve at 31 mS⋅cm^−1^, representing total quantity of pure motor domain isolated.

### Transient and steady-state kinetic measurements

Steady-state ATPase activity was measured using an NADH-coupled enzyme assay in the following reaction buffer: 10-mM Mops (pH 7.0), 5-mM MgCl_2_, 0.1-mM EGTA, 10-mM KCl, 2-mM MgATP, 40 U mL^−1^ lactate dehydrogenase, 200 U mL^−1^ pyruvate kinase, 1-mM phosphoenolpyruvate, and 200-μM NADH. The time course of NADH absorbance at A_340_ was measured using a UV-1800 dual-beam spectrophotometer (Shimadzu) with a multicell cuvette at a constant 20 ± 0.1 °C. The rate of ATP consumption (*i.e.*, ADP production) was calculated from the change in NADH using ε = 6220 M^−1^ cm^−1^. The final concentration of myosin in each reaction was 30 nM.

Transient kinetic analysis was performed using a HiTech SF61-DX2 dual-mixing stopped-flow spectrophotometer (TgK Scientific) or RQF-3 quenched-flow (Kintek). Stopped-flow excitation wavelength and emission filters were as follows: intrinsic protein fluorescence (297 nm, WG320), pyrene-actin (365 nm, GG400), MDCC-PbP (430 nm, LP450), and mant-nucleotides (297 nm, GG400). Orthogonal light scattering was measured at 340 nm without emission filtering. Concentrated stocks of actin and myosin were pretreated using 0.02 U/ml apyrase for 30 min at room temperature to remove trace ATP and ADP contamination. After dilution to the final experimental conditions, apyrase was <0.001 U mL^−1^. For phosphate release experiments, the stopped-flow apparatus and all solutions were incubated with 7-methylguanosine (0.5 mM) and nucleoside phosphorylase (0.02 U mL^−1^) to scavenge contaminating P_i_. The fluorescent phosphate-binding probe, MDCC-PbP, was included in all reactants at a final concentration of 5 μM. The volume ratio for single mixing stopped-flow reactions was 1:1. For double-mixing phosphate release measurements, ATP and EGFP-M15-2IQ were mixed at a 1:1 ratio, aged in a delay loop, before mixing 1:1 with actin. The final ratio of ATP, EGFP-M15-2IQ, and actin in the observation cell was 1:1:2 (vol/vol), respectively. Quenched-flow analysis was performed as previously described (47). In general, all reactions were mixed under pseudo–first-order conditions, with one reactant in > tenfold excess, unless otherwise stated. Rate and equilibrium constants were extracted from experimental data using established analytical approaches (44, 53, 104). All reactions were performed in 20-mM Mops (pH 7.0), 100-mM KCl, 5-mM MgCl_2_, and 0.1-mM EGTA at 20 ± 0.1 °C.

### Data analysis

Nonlinear least-squares regression was performed using Kinetic Studio (TgK Scientific) and Prism 8 (GraphPad). Uncertainties arising from nonlinear regression are reported as the SEM and for all other measurements as SD. Uncertainties were propagated for parameters that required multiplication or division of primary experimental values. Experimental data are from three independent experiments, using at least two independent myosin preparations, unless otherwise stated.

## Data availability

All data referenced are contained within the article. The *Sf*9–UNC45A and *Sf*9–UNC45B cell lines are available from Jonathan E. Bird.

## Conflict of interest

The authors declare that they have no conflicts of interest with the contents of this article.
